# Genetic-and-Epigenetic Interspecies Networks for Cross-Talk Mechanisms in Human Macrophages and Dendritic Cells during MTB Infection

**DOI:** 10.3389/fcimb.2016.00124

**Published:** 2016-10-18

**Authors:** Cheng-Wei Li, Yun-Lin Lee, Bor-Sen Chen

**Affiliations:** Laboratory of Control and Systems Biology, National Tsing Hua UniversityHsinchu, Taiwan

**Keywords:** genetic and epigenetic interspecies network, macrophages, dendritic cells, *Mycobacterium tuberculosis* infection, cross-talk mechanism, multi-molecule drug, potential drug targets, principal network projection (PNP)

## Abstract

Tuberculosis is caused by *Mycobacterium tuberculosis* (*Mtb*) infection. *Mtb* is one of the oldest human pathogens, and evolves mechanisms implied in human evolution. The lungs are the first organ exposed to aerosol-transmitted *Mtb* during gaseous exchange. Therefore, the guards of the immune system in the lungs, such as macrophages (Mϕs) and dendritic cells (DCs), are the most important defense against *Mtb* infection. There have been several studies discussing the functions of Mϕs and DCs during *Mtb* infection, but the genome-wide pathways and networks are still incomplete. Furthermore, the immune response induced by Mϕs and DCs varies. Therefore, we analyzed the cross-talk genome-wide genetic-and-epigenetic interspecies networks (GWGEINs) between Mϕs vs. *Mtb* and DCs vs. *Mtb* to determine the varying mechanisms of both the host and pathogen as it relates to Mϕs and DCs during early *Mtb* infection. First, we performed database mining to construct candidate cross-talk GWGEIN between human cells and *Mtb*. Then we constructed dynamic models to characterize the molecular mechanisms, including intraspecies gene/microRNA (miRNA) regulation networks (GRNs), intraspecies protein-protein interaction networks (PPINs), and the interspecies PPIN of the cross-talk GWGEIN. We applied a system identification method and a system order detection scheme to dynamic models to identify the real cross-talk GWGEINs using the microarray data of Mϕs, DCs and *Mtb*. After identifying the real cross-talk GWGEINs, the principal network projection (PNP) method was employed to construct host-pathogen core networks (HPCNs) between Mϕs vs. *Mtb* and DCs vs. *Mtb* during infection process. Thus, we investigated the underlying cross-talk mechanisms between the host and the pathogen to determine how the pathogen counteracts host defense mechanisms in Mϕs and DCs during *Mtb* H37Rv early infection. Based on our findings, we propose Rv1675c as a potential drug target because of its important defensive role in Mϕs. Furthermore, the membrane essential proteins v1098c, and Rv1696 (or cytoplasm for Rv0667), in *Mtb* could also be potential drug targets because of their important roles in *Mtb* survival in both cell types. We also propose the drugs Lopinavir, TMC207, ATSM, and GTSM as potential therapeutic treatments for *Mtb* infection since they target the above potential drug targets.

## Introduction

Tuberculosis (TB) is an ancient disease of humankind, accounting for a significant number of deaths over the years. According to the World Health Organization (WHO) Global Health Observatory (GHO) data for 2014, approximately 9.6 million new cases of TB are identified each year, with almost 1.5 million TB related deaths. Approximately one-third of the global population harbor this bacterium but remain asymptomatic, also known as latent TB (Dye et al., 1999). Of those with latent TB, only 5–10% will develop into active TB disease in their lifetime (Vynnycky and Fine, 2000). The main etiologic agent of TB is *Mycobacterium tuberculosis* (*Mtb*), which was first identified as a pathogen by Robert Koch in 1882 (Koch, 1982).

Dendritic cells (DCs), which are involved in the first line of immune defense, protect tissues from extraneous bacilli or viral infection. DCs also phagocytose *Mtb*, process the bacilli, and present the mycobacterial antigens on the plasma membrane. The DCs migrate to the regional lymph nodes where they prime T cells by presenting antigens on MHC and secrete cytokines. DCs in peripheral tissue capture and process antigens in different ways: (1) macropinocytosis (Sallusto et al., 1995), (2) endocytosis via receptors such as the mannose receptor DEC-205 or dendritic cell specific ICAM 3 grabbing non-integrin (DC-SIGN) (Geijtenbeek et al., 2000), (3) Fc receptors and complement receptors (CR3), which mediate efficient internalization of immune complexes or bacteria (Rescigno et al., 2000), (4) phagocytosis of viruses or bacteria (Albert et al., 1998), (5) TLR-mediated pathogen recognition (Visintin et al., 2001).

The interactions between DCs and *Mtb* are still not fully understood and some reports are contradictory. For instance, after interacting with pathogens, DCs mature and migrate to lymph nodes where they prime T cells by presenting antigens on MHC and secrete cytokines such as IL-12 (Lipscomb and Masten, 2002). In contrast, a study reported that interaction of *Mtb* with TLRs induce high IL-12 secretion during early infection whereas interaction with DC-SIGN results in high IL-10 secretion (Kaufmann and Schaible, 2003).

After *Mtb* is phagocytosed by alveolar macrophages (Mϕs), ligation of TLR2 and TLR4 stimulates the secretion of proinflammatory cytokines such as TNF-α, IL-1β, and IL-12 (Means et al., 1999). TNF-α plays an important role in the immune response to *Mtb* infection by stimulating neutrophils and Mϕs in an autocrine and paracrine fashion to induce apoptosis and production of reactive nitrogen intermediates (RNIs). Nitric oxide, an iNOS product, is highly toxic to intracellular mycobacteria (Chan et al., 1992). NOS_2_-deficient mice showed increased susceptibility to mycobacterial infection (Garcia et al., 2000). Some studies reported that TNF-α is important in preventing the reactivation of latent TB in non-human primate and mouse models (Scanga et al., 1999; Lin et al., 2010). IL-1β was correlated to disease activity and fever in infected patients (Tsao et al., 1999). IL-12 is important in driving T helper type 1 (Th1) differentiation and IFN-γ production. The increased susceptibility of IL-12p40 gene deficient mice to *Mtb* infection further supports an important role for IL-12 in the protective immune response against *Mtb* (Cooper et al., 1997). However, IFN-γ is the most important cytokine in the immune response to mycobacteria, and it plays a role in activating Mϕs to produce reactive oxygen and nitrogen species. IFN-γ deficient mice are highly susceptible to *Mtb* infection and produce less NOS_2_ (Flynn et al., 1993).

The host immune response is often insufficient for handling *Mtb* infection as the bacterium has developed sophisticated defense mechanisms such as blocking maturation, lysosomal fusion, and acidification to survive in Mϕs and enhance the growth of bacteria. The 19-kDa lipoprotein of *Mtb* interacts with host antigen presenting cells (APCs) via TLR1/2 to reduce antigen processing and MHC-II expression (Noss et al., 2001), rather than inhibiting cytokine production (Sugawara et al., 2003). ESAT-6 has a similar effect through TLR-2 (Pathak et al., 2007). Lipoarabinomannan (LAM) is a major cell wall component of *Mtb* that binds to DC-SIGN. The binding of LAM inhibits DC maturation, decreases IL-12 production, and induces DCs to secrete IL-10 (van Kooyk and Geijtenbeek, 2003). IL-10 is an immune suppressive cytokine, and its induction by *Mtb* allows survival of the bacteria (Redford et al., 2011). Blocking the accumulation of ATPases and GTPases in the vacuole interferes with the function of the phagosome by decreasing the pH needed to kill the bacteria (Sturgill-Koszycki et al., 1994). Other mechanisms of Mtb immune evasion have been thoroughly outlined in a previous review (Sakamoto, 2012).

Although, a few studies investigated the effects of human microRNAs (miRNAs) on *Mtb*-infected Mϕ (Rajaram et al., 2011; Kumar et al., 2012) and DC (Chatterjee et al., 2011; Singh et al., 2013), it is still a big issue to identify the cross-talk genome-wide genetic-and-epigenetic interspecies networks (GWGEINs) between pathogen and host both in Mϕs and DCs during *Mtb* infection. Additionally, the different offensive and defensive mechanisms between Mϕs vs. *Mtb* and DCs vs. *Mtb* play important roles in the evaluation of potential drugs for treating human cells during early *Mtb* infection. In this study, we identified the cross-talk GWGEINs in both human Mϕs and DCs during early *Mtb* infection through systems biology approach. We proposed the common and different pathways of the host-and-pathogen core networks (HPCNs), extracted from the cross-talk GWGEINs, to investigate the cellular mechanisms of both host and pathogen in Mϕs and DCs during early *Mtb* infection. Moreover, we also discussed the cross-talks between host and pathogen and inferred the core network biomarkers to get an insight into offensive and defensive mechanisms both in Mϕs vs. *Mtb* and DCs vs. *Mtb*. Finally, based on the core network biomarkers of offensive and defensive mechanisms between Mϕs, DCs and *Mtb*, we proposed the potential multiple drug targets and suggested the potential multi-molecule drug for treating human cells during early *Mtb* infection.

## Materials and methods

### Overview of the construction processes of cross-talk GWGEINs in Mϕs and DCs infected with *Mtb*

**Figure 1:**
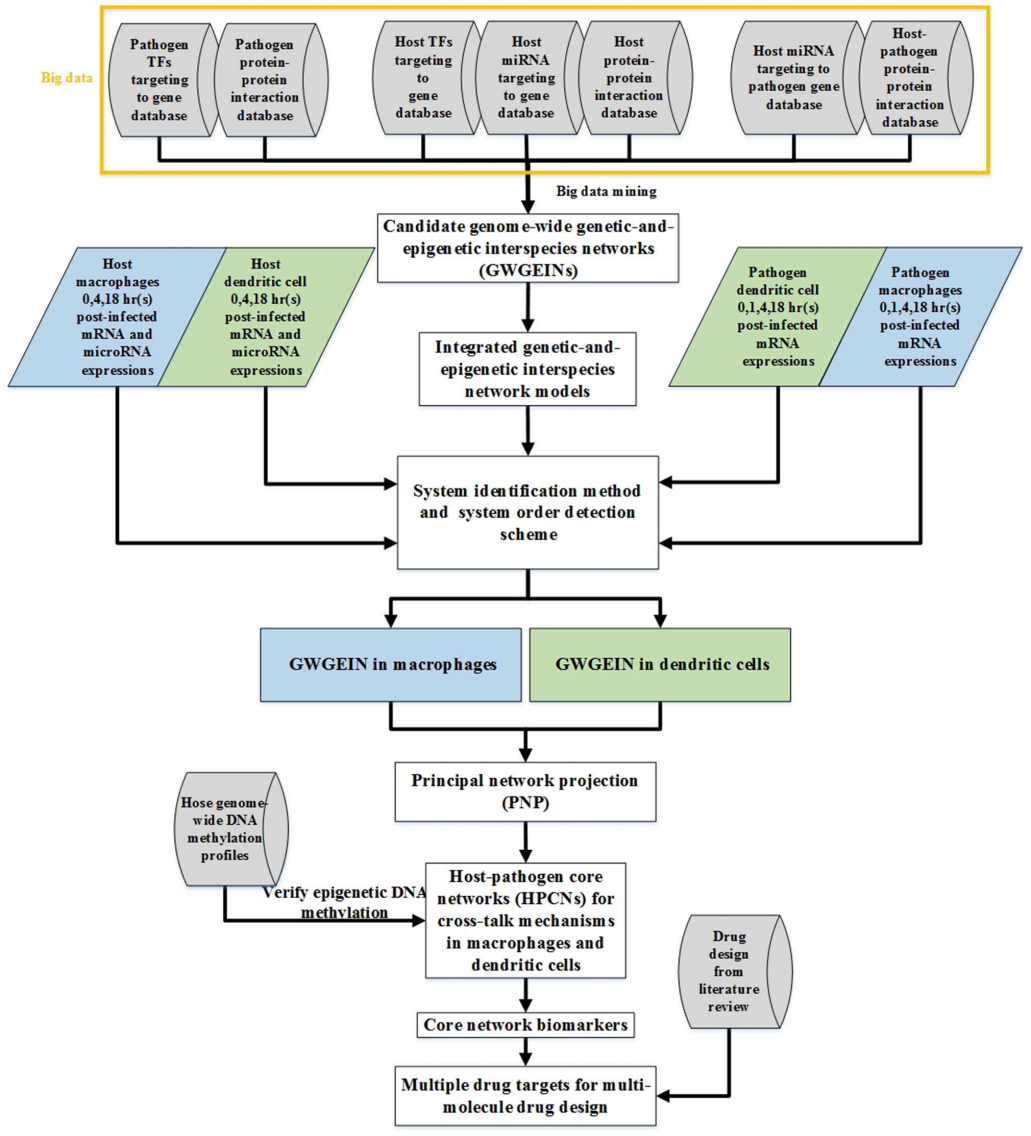
**Flow chart of networks constructed using the systems biology approach**. The construction of candidate GWGEINs requires several association databases as Cylinder blocks. We used network models and genome-wide high throughput data to identify the regulative and interactive abilities in candidate GWGEINs, and pruned the false-positives of candidate GWGEINs by deleting the insignificant components out of system order as determined by Akaike information criterion (AIC). Then GWGEINs in Mϕs and DCs were constructed to represent the real cellular network of biological systems. Finally, we extracted host-pathogen core networks (HPCNs) from GWGEINs by principal network projection (PNP) to investigate the defensive mechanisms of the host and pathogen in Mϕs and DCs during *Mtb* infection. The multiple drug targets are also selected based on core network markers of HPCNs for potential multi-molecule drug design through drug design literature search.

The flowchart of the strategy for constructing cross-talk GWGEINs and the HPCNs in Mϕs and DCs during early *Mtb* infection is shown in Figure 1. The cross-talk GWGEIN comprises host/pathogen gene/miRNA regulation networks (GRNs), host/pathogen protein-protein interaction networks (PPINs), the interspecies PPIN and the regulation networks of host-miRNAs on host-/pathogen-genes. The constructions of GWGEIN and HPCN can be divided into three steps: (1) Big data mining and data preprocessing for candidate cross-talk GWGEINs, (2) the identification of the real cross-talk GWGEIN by applying the system identification method and the system order detection scheme using the genome-wide microarray data of Mϕs, DCs, and *Mtb* during early *Mtb* infection, (3) HPCN construction by applying principal network projection (PNP) to the real cross-talk GWGEIN. This allowed identification of the differential cross-talk mechanisms between Mϕs and DCs during early *Mtb* infection.

### Big data mining and data preprocessing

In this study, in order to identify the real cross-talk GWGEIN between human cells and *Mtb*, the simultaneously measured activities of host and pathogen during infection process were required. Since several gene expression profiles are available only on the host side (Realegeno et al., 2016) or only on the pathogen side (Fontán et al., 2008) during *Mtb* infection, those data was not suitable for identifying the offensive and defensive mechanisms between host and pathogen during infection process. Therefore, in this study, the microarray raw data, obtained from a previous study investigating Mϕs and DCs infected with *Mtb* (Tailleux et al., 2008), were the only one that presents all the data necessary to build the model of the cross-talk GWGEIN. This issue was also a limitation of the model as well. These assumptions should be revised as more data becomes available.

The raw data has two parts. The first part includes the mRNA and miRNA expression levels of human Mϕs and DCs at 0, 4, 18, and 48 h post-infection with *Mtb* H37Rv (ArrayExpress accession number E-MEXP-3521). The second part includes the *Mtb* transcriptional mRNA expression levels in Mϕs and DCs at 0, 1, 4, and 18 h post infection (ArrayExpress accession number E-BUGS-58; http://www.ebi.ac.uk/arrayexpress/experiments/browse.html). The probing platforms used in the host and pathogen were Affymetrix GeneChip Human Genome HG-U133A and *M. tuberculosis*/*M. bovis* 10944 YBv2_1_1, respectively, which contained 22283 and 10944 probes, respectively. The data of human Mϕs at 0, 4, 18 h and *Mtb* at 0, 1, 4, 18 h during infection process were employed to identify the real cross-talk GWGEIN between Mϕs and *Mtb*, while the data of human DCs at 0, 4, 18 h and *Mtb* at 0, 1, 4, 18 h during infection process were employed to identify the real cross-talk GWGEIN between DCs and *Mtb*. In fact, we used cubic spline to obtain the sufficient number of data points for applying system identification method. Therefore, in human Mϕs and DCs, we applying cubic spline to the data at 0, 4, 18, and 48 h to obtain the sufficient number of data points between 0 and 18 h to avoid overfitting problem in parameter identification process.

Constructing the candidate cross-talk GWGEIN requires big data obtained from experimental or computational predictions. The big data, which collected from several databases, were as follows. The host candidate GRN required miRNA for regulatory gene associations from TargetScanHuman (Agarwal et al., 2015), transcription factors (TFs) to regulatory gene associations from GSEA (Mootha et al., 2003; Subramanian et al., 2005), HTRIdb (Bovolenta et al., 2012), and ITFP (Zheng et al., 2008). The host candidate PPIN need protein-protein interaction (PPI) associations from BioGRID (Stark et al., 2006). The pathogen candidate GRN required TF for gene regulatory associations from TbDb (Galagan J. et al., 2013; Galagan J. E. et al., 2013; Jaini et al., 2014), the data in Minch et al. (2015) and the host-miRNAs targeting pathogen-gene associations in Guo et al. (2010). The pathogen candidate PPIN required PPI associations from STRING (Szklarczyk et al., 2015), and the study in Wang et al. (2010). The host-pathogen candidate PPIN required interspecies PPI associations from the study in Davis et al. (2007) and Zhou et al. (2014).

In order to support inferred epigenetic DNA methylation, we used the genome-wide DNA methylation profiles of monocytes (GSE70478) (Esterhuyse et al., 2015) and DCs (GSE64177) (Pacis et al., 2015) infected with *Mtb* (with sample size 10 and 6, respectively). According to the DNA methylation analysis of monocytes and macrophages, only 27 CpG sites displayed differential DNA methylation during the maturation step (Vento-Tormo et al., 2016). Therefore, the DNA methylation analysis between monocytes and DCs could represent that between Mϕs and DCs. Methylation data were analyzed using one-way analysis of variance (ANOVA) statistics.

To integrate the above big data, including genome-wide expression data, the data for constructing the candidate cross-talk GWGEIN, and genome-wide DNA methylation profiles, we used Matlab's text-file and string manipulation tools in text mining to unify the gene name based on the gene symbols in the National Center for Biotechnology Information's (NCBI's) Gene database.

Therefore, in host candidate GRN, we obtained 445,335 TF gene pairs and 411,418 miRNA gene pairs. In the databases of pathogen candidate GRN, we obtained 85,916 TF gene pairs and 12 miRNA gene pairs. In the databases of interspecies candidate PPIN, we obtained 1,101,388 host PPI pairs, 1868 pathogen PPI pairs, and 10,536 host-pathogen interspecies PPI pairs. Finally, we integrated the candidate pairs into the candidate cross-talk GWGEIN (Figure 1). The candidate cross-talk GWGEIN was then used to identify the real cross-talk GWGEINs in Mϕs and DCs by applying the system identification method and the system order detection scheme using the genome-wide microarray data of Mϕs, DCs and *Mtb* during early *Mtb* infection.

### Dynamic models of the cross-talk GWGEIN for Mϕs, DCs, and *Mtb* during early infection process

Because all connections in the candidate GWGEIN were obtained from a number of plausible predictions in human cells and *Mtb*, we then constructed the dynamic models of GWGEIN, which characterize the molecular mechanisms in GWGEIN, to prune false-positive connections using genome-wide microarray data and finally obtained the real cross-talk GWGEIN between Mϕs and *Mtb* (or DCs and *Mtb*) during infection process.

For the GRN of host genes in GWGEIN, the dynamic model of the *i*th host-gene can be described as the following stochastic dynamic equation,

(1) $$\begin{array}{l} \begin{array}{c} x_{i}\;\left(t+1\right)=x_{i}\left(t\right)+\overset{J_{i}}{\underset{j=1}{\sum }}a_{ij}y_{j}\left(t\right)-\overset{K_{i}}{\underset{k=1}{\sum }}c_{ik}x_{i}\left(t\right)miRNA_{k}\left(t\right)\\ \;-\lambda_{i}x_{i}\left(t\right)+\delta_{i}+\omega_{i}\left(t\right),\text{for }i=1,\cdots \;,I \end{array} \end{array}$$

where *x*_*i*_(*t*), *y*_*j*_(*t*), and *miRNA*_*k*_(*t*) represent the expression levels of the *i*th host-gene, the *j*th host-TF, and the *k*th host-miRNA at time *t*, respectively; *a*_*ij*_ and −*c*_*ik*_ denote the abilities of the *j*th host-TF regulation and the *k*th host-miRNA repression on the *i*th host-gene; δ_*i*_ and −λ_*i*_ indicate the basal level and the degradation rate of the *i*th host-gene, respectively; *J*_*i*_ and *K*_*i*_ signify the numbers of host TFs and miRNAs regulating the *i*th host-gene in the candidate GWGEIN; and ω_*i*_(*t*) is the stochastic noise of the *i*th host-gene due to modeling residue. The dynamic model of host genes in (1) characterized molecular mechanisms, including the transcription regulations by $\overset{J_{i}}{\underset{j=1}{\sum }}a_{ij}y_{j}\left(t\right)$, the miRNA repressions by $-\overset{K_{i}}{\underset{k=1}{\sum }}c_{ik}x_{i}\left(t\right)$*miRNA*_*k*_(*t*), the mRNA degradation by −λ_*i*_*x*_*i*_(*t*), the basal level by δ_*i*_, and the stochastic noise by ω_*i*_(*t*). Owing to the direct effects of DNA methylation on the binding affinities of RNA polymerase to target genes (Weber et al., 2007), we assumed that the change of basal level δ_*i*_ between the *Mtb*-infected Mϕs and the *Mtb*-infected DCs in the dynamic model (1) indicates the occurrence of methylation on the *i*th host-gene.

For the PPIN of host proteins in GWGEIN, the dynamic model of the *j*th host-protein can be described as the following stochastic dynamic equation,

(2) $$\begin{array}{l} y_{j}\left(t+1\right)=y_{j}\left(t\right)+\overset{J_{j}}{\underset{l=1}{\sum }}b_{jl}y_{j}\left(t\right)y_{l}\left(t\right)+\overset{M_{j}}{\underset{m=1}{\sum }}d_{jm}y_{j}\left(t\right)w_{m}\left(t\right)\\ +\alpha_{j}x_{j}\left(t\right)-\gamma_{j}y_{j}\left(t\right)+\kappa_{j}+\varpi_{j}\left(t\right),\text{ for }j=1,\cdots \;,J \end{array}$$

where *y*_*j*_(*t*), *y*_*l*_(*t*), *x*_*j*_(*t*), and *w*_*m*_(*t*) represent the expression levels of the *j*th host-protein, the *l*th host-protein, the *j*th host-gene and the *m*th pathogen-protein at time *t*, respectively; *b*_*jl*_ and *d*_*jm*_ denote the interactive abilities between the *j*th host-protein and *l*th host-protein and between the *j*th host-protein and the *m*th pathogen-protein, respectively; α_*j*_, −γ_*j*_, and κ_*j*_ indicate the translation rate, the degradation rate and the basal level of the *j*th host-protein; *J*_*j*_ and *M*_*j*_ signify the numbers of host proteins and pathogen proteins interacting with the *j*th host-protein in the candidate GWGEIN; and ${\bar{\omega }}_{j}\left(t\right)$ is the stochastic noise due to modeling residue. The dynamic model of host proteins in (2) characterized molecular mechanisms, including the intraspecies host PPIs by $\overset{J_{j}}{\underset{l=1}{\sum }}b_{jl}y_{j}\left(t\right)y_{l}\left(t\right)$, the interspecies PPIs by $\overset{M_{j}}{\underset{m=1}{\sum }}d_{jm}y_{j}\left(t\right)w_{m}\left(t\right)$, the protein translation by α_*j*_x_*j*_(*t*), the protein degradation by −γ_*j*_y_*j*_(*t*), the basal level by κ_*j*_, and the stochastic noise by ω_*j*_(*t*).

For the GRN of pathogen genes in GWGEIN, the dynamic model of the *n*th pathogen-gene can be described as the following stochastic dynamic equation,

(3) $$\begin{array}{l} v_{n}\left(t+1\right)=v_{n}\left(t\right)+\overset{M_{n}}{\underset{m=1}{\sum }}e_{nm}w_{m}\left(t\right)-\overset{K_{n}}{\underset{k=1}{\sum }}g_{nk}v_{n}\left(t\right)miRNA_{k}\left(t\right)\\ -\;\rho_{n}v_{n}\left(t\right)+\eta_{n}+\psi_{n}\left(t\right),\\ \text{for }n=1,\cdots \;,N \end{array}$$

where *v*_*n*_(*t*) represents the expression level of the *n*th pathogen-gene at time *t*; *e*_*nm*_ and −*g*_*nk*_ denote the abilities of the *m*th pathogen-TF regulation and the *k*th host-miRNA repression on the *n*th pathogen-gene, respectively; η_*n*_ and −ρ_*n*_ indicate the basal level and the degradation rate of the *n*th pathogen-gene, respectively; *M*_*n*_ and *K*_*n*_ signify the numbers of pathogen TFs and host miRNAs regulating the *n*th pathogen-gene in the candidate GWGEIN; and ψ_*n*_(*t*) is the stochastic noise due to modeling residue. The dynamic model of pathogen genes in (3) characterized molecular mechanisms, including the pathogen transcription regulations by $\overset{M_{n}}{\underset{m=1}{\sum }}e_{nm}w_{m}\left(t\right)$, the host miRNA repressions by $-\overset{K_{n}}{\underset{k=1}{\sum }}g_{nk}v_{n}\left(t\right)miRNA_{k}\left(t\right)$,the pathogen mRNA degradation by −ρ_*n*_*v*_*n*_(*t*), the pathogen basal level by η_*n*_, and the stochastic noise by ψ_*n*_(*t*). Because it has been proposed that miRNAs can exist extracellularly and circulate in body fluid (Weber et al., 2010; Liu et al., 2016), we considered the repressions of host-miRNAs on pathogen-genes in the dynamic model (3) of GWGEIN.

For the PPIN of pathogen proteins in GWGEIN, the dynamic model of the *m*th pathogen-protein can be described as the following stochastic dynamic equation,

(4) $$\begin{array}{lll} w_{m}\left(t+1\right) & = & w_{m}\left(t\right)+\overset{M_{m}}{\underset{q=1}{\sum }}h_{mq}w_{m}\left(t\right)w_{q}\left(t\right)+\overset{J_{m}}{\underset{j=1}{\sum }}d_{mj}w_{m}\left(t\right)y_{j}\left(t\right)\\ +\beta_{m}v_{m}\left(t\right)-\sigma_{m}w_{m}\left(t\right)+\varepsilon_{m}+\tau_{m}\left(t\right),\\ \text{for }m=1,\cdots \;,M \end{array}$$

where *w*_*q*_(*t*) and *w*_*m*_(*t*) represent the expression level of the *q*th pathogen-protein and the *m*th pathogen-protein at time *t*; *h*_*mq*_ and *d*_*mj*_ denote the interactive abilities between the *m*th pathogen-protein and *q*th pathogen-protein and between the *m*th pathogen-protein and the *j*th host-protein, respectively; β_*m*_, −σ_*m*_, and ε_*m*_ indicate the translation rate from the mRNA of the *m*th pathogen-gene *v*_*m*_, and the degradation rate and the basal level of the *m*th pathogen-protein; *M*_*m*_ and *J*_*m*_ signify the numbers of pathogen proteins and host proteins interacting with the *m*th pathogen-protein in the candidate GWGEIN; and τ_*m*_(*t*) is the stochastic noise due to modeling residue. The dynamic model of pathogen proteins in (4) characterized molecular mechanisms, including the intraspecies pathogen PPIs by $\overset{M_{m}}{\underset{q=1}{\sum }}h_{mq}w_{m}\left(t\right)w_{q}\left(t\right)$, the interspecies PPIs by $\overset{J_{m}}{\underset{j=1}{\sum }}d_{mj}w_{m}\left(t\right)y_{j}\left(t\right)$, the protein translation by β_*m*_v_*m*_(*t*), the protein degradation by -σ_*m*_w_*m*_(*t*), the basal level by ε_*m*_, and the stochastic noise by τ_*m*_(*t*).

### System identification method of the dynamic models of GWGEIN

After constructing the stochastic dynamic Equations (1–4) in the GWGEIN, we applied a system identification method to the Equations (1)–(4) to identify their respective parameters. The host GRN Equation (1) was rewritten as the following linear regression form,

(5) $$\begin{array}{l} x_{i}(t+1)=[\begin{array}{cccc} y_{1}(t) & \cdots & y_{J_{i}}(t) & x_{i}(t)miRNA_{1}(t) \end{array}\\ \begin{array}{cccc} \cdots & x_{i}(t)miRNA_{K_{i}}(t) & x_{i}(t) & 1 \end{array}]\left[\begin{array}{c} a_{i1}\\ \vdots \\ a_{iJ_{i}}\\ \begin{array}{c} -c_{i1}\\ \begin{array}{c} \begin{array}{c} \vdots \\ -c_{iK_{i}} \end{array}\\ \begin{array}{c} 1-\lambda_{i}\\ \delta_{i} \end{array} \end{array} \end{array} \end{array}\right]+\omega_{i}(t) \end{array}$$

which could be simply represented as follows,

(6) $$x_{i}(t+1)=\phi_{i}^{HG}(t)\theta_{i}^{HG}+\omega_{i}(t),\text{ for }i=1{,}^{\ldots },I$$

where $\Phi_{i}^{HG}\left(t\right)$ denotes the regression vector obtained from the corresponding expression data; and $\theta_{i}^{HG}$ represents the unknown parameter vector of the host gene *i* in the host GRN to be estimated.

The Equation (6) can be augmented for *F*_*i*_ time points of the *i*th host-gene as follows,

(7) $$\begin{array}{l} \left[\begin{array}{c} x_{i}\left(t_{2}\right)\\ x_{i}\left(t_{3}\right)\\ \vdots \\ x_{i}\left(t_{F_{i}}+1\right) \end{array}\right]=\left[\begin{array}{c} \phi_{i}^{HG}\left(t_{1}\right)\\ \phi_{i}^{HG}\left(t_{2}\right)\\ \vdots \\ \phi_{i}^{HG}\left(t_{F_{i}}\right) \end{array}\right]\theta_{i}^{HG}+\left[\begin{array}{c} \omega_{i}\left(t_{1}\right)\\ \omega_{i}\left(t_{2}\right)\\ \vdots \\ \omega_{i}\left(t_{F_{i}}\right) \end{array}\right] \end{array}$$

For simplicity, we defined the notations ${\text{X}}_{i}^{HG}$, $\Phi_{i}^{HG}$, and $\Gamma_{i}^{HG}$ to represent (7) as follows,

(8) $$\begin{array}{lll} {\text{X}}_{i}^{HG} & = & \Phi_{i}^{HG}\theta_{i}^{HG}+\Gamma_{i}^{HG}\\ \text{where }{\text{X}}_{i}^{HG} & = & \left[\begin{array}{c} x_{i}\left(t_{2}\right)\\ x_{i}\left(t_{3}\right)\\ \vdots \\ x_{i}\left(t_{F_{i}}+1\right) \end{array}\right],\Phi_{i}^{HG}=\left[\begin{array}{c} \phi_{i}^{HG}\left(t_{1}\right)\\ \phi_{i}^{HG}\left(t_{2}\right)\\ \vdots \\ \phi_{i}^{HG}\left(t_{F_{i}}\right) \end{array}\right],\text{and}\\ \Gamma_{i}^{HG} & = & \left[\begin{array}{c} \omega_{i}\left(t_{1}\right)\\ \omega_{i}\left(t_{2}\right)\\ \vdots \\ \omega_{i}\left(t_{F_{i}}\right) \end{array}\right]. \end{array}$$

The system identification method of the host GRN Equation (1) can then be formulated by the following constrained least square parameter estimation problem,

(9) $$\begin{array}{l} \underset{\theta_{i}^{HG}}{\min}\frac{1}{2}{\left\|\Phi_{i}^{HG}\theta_{i}^{HG}-{\text{X}}_{i}^{HG}\right\|}_{2}^{2}\\ \text{Subject to}\overset{J_{i}}{\overset{︷}{[\begin{array}{ccc} 0 & \cdots & 0\\ \vdots & \ddots & \vdots \\ 0 & \cdots & 0\\ 0 & \cdots & 0\\ 0 & \cdots & 0 \end{array}}}\overset{K_{i}}{\overset{︷}{\begin{array}{ccc} 1 & \cdots & 0\\ \vdots & \ddots & \vdots \\ 0 & \cdots & 1\\ 0 & \cdots & 0\\ 0 & \cdots & 0 \end{array}}}\begin{array}{cc} 0 & 0\\ \vdots & \vdots \\ 0 & 0\\ 1 & 0\\ 0 & -1 \end{array}]\theta_{i}^{HG}\leq \left[\begin{array}{c} 0\\ \vdots \\ 0\\ 1\\ 0 \end{array}\right] \end{array}$$

By solving the problem in (9), we can obtain the parameters in the host GRN Equation (1) and simultaneously guarantee the non-positive host-miRNA repression −*c*_*ik*_ ≤ 0, the non-positive host-gene degradation −λ_*i*_ ≤ 0, and the non-negative host-gene basal level δ_*i*_ ≥ 0.

Similarly, the host PPIN Equation (2) can be rewritten in the following linear regression form,

(10) $$\begin{array}{l} y_{j}(t+1)=[\begin{array}{llll} y_{j}(t)y_{1}(t) & \cdots & y_{j}(t)y_{J_{j}}(t) & y_{j}(t)w_{1}(t) \end{array}\\ \;\begin{array}{lllll} \cdots & y_{j}(t)w_{M_{j}}(t) & x_{j}(t) & y_{j}(t) & 1 \end{array}]\left[\begin{array}{c} b_{j1}\\ \vdots \\ b_{jJ_{j}}\\ \begin{array}{c} d_{j1}\\ \begin{array}{c} \begin{array}{c} \vdots \\ d_{jM_{j}} \end{array}\\ \begin{array}{c} \alpha_{j}\\ \begin{array}{c} 1-\gamma_{j}\\ \kappa_{j} \end{array} \end{array} \end{array} \end{array} \end{array}\right]+\varpi_{j}(t) \end{array}$$

which could be simply represented as follows,

(11) $$\begin{array}{l} y_{j}\left(t+1\right)=\phi_{j}^{HP}\left(t\right)\theta_{j}^{HP}+\varpi_{j}\left(t\right),for\;j=1{,}^{\ldots },J \end{array}$$

where $\Phi_{j}^{HP}\left(t\right)$ denotes the regression vector obtained from the corresponding expression data; and $\theta_{j}^{HP}$ represents the unknown parameter vector of the host protein *j* in the host PPIN to be estimated. The Equation (11) can be augmented for *F*_*j*_ time points of the *j*th host-protein as follows,

(12) $$\begin{array}{lll} {\text{Y}}_{j}^{HP} & = & \Phi_{j}^{HP}\theta_{j}^{HP}+\Gamma_{j}^{HP}\\ \text{where }{\text{Y}}_{j}^{HP} & = & \left[\begin{array}{c} y_{j}\left(t_{2}\right)\\ y_{j}\left(t_{3}\right)\\ \vdots \\ y_{j}\left(t_{F_{j}}+1\right) \end{array}\right]\text{,}\Phi_{j}^{HP}=\left[\begin{array}{c} \phi_{j}^{HP}\left(t_{1}\right)\\ \phi_{j}^{HP}\left(t_{2}\right)\\ \vdots \\ \phi_{j}^{HP}\left(t_{F_{j}}\right) \end{array}\right]\text{, and}\\ \Gamma_{j}^{HP} & = & \left[\begin{array}{c} \varpi_{j}\left(t_{1}\right)\\ \varpi_{j}\left(t_{2}\right)\\ \vdots \\ \varpi_{j}\left(t_{F_{j}}\right) \end{array}\right]. \end{array}$$

The system identification method of the host PPIN Equation (2) can then be formulated by the following constrained least square parameter estimation problem,

(13) $$\begin{array}{l} \underset{\theta_{j}^{HP}}{\min}\frac{1}{2}{\left\|\Phi_{j}^{HP}\theta_{j}^{HP}-{\text{Y}}_{j}^{HP}\right\|}_{2}^{2}\\ \text{Subject to}\overset{J_{j}+M_{j}}{\overset{︷}{[\begin{array}{ccc} 0 & \cdots & 0\\ 0 & \cdots & 0\\ 0 & \cdots & 0 \end{array}}}\begin{array}{ccc} -1 & 0 & 0\\ 0 & 1 & 0\\ 0 & 0 & -1 \end{array}]\theta_{j}^{HP}\leq \left[\begin{array}{c} 0\\ 1\\ 0 \end{array}\right] \end{array}$$

By solving the problem in (13), we can obtain the parameters in the host PPIN Equation (2) and simultaneously guarantee the non-negative host-protein coding rate α_*j*_ ≥ 0, the non-positive host-protein degradation −γ_*j*_ ≤ 0, and the non-negative host-protein basal level κ_*j*_ ≥ 0.

As the same process in host GRN Equation (5), the pathogen GRN Equation (3) can be rewritten as follows,

(14) $$\begin{array}{l} v_{n}(t+1)=[\begin{array}{llll} w_{1}(t) & \cdots & w_{M_{n}}(t) & v_{n}(t)miRNA_{1}(t) \end{array}\\ \begin{array}{llll} \cdots & v_{n}(t)miRNA_{K_{n}}(t) & v_{n}(t) & 1 \end{array}]\left[\begin{array}{c} e_{n1}\\ \vdots \\ e_{nM_{n}}\\ \begin{array}{c} -g_{n1}\\ \begin{array}{c} \begin{array}{c} \vdots \\ -g_{nK_{n}} \end{array}\\ \begin{array}{c} 1-\rho_{n}\\ \eta_{n} \end{array} \end{array} \end{array} \end{array}\right]+\psi_{n}(t) \end{array}$$

which could be simply represented as follows,

(15) $$\begin{array}{l} v_{n}\left(t+1\right)=\phi_{n}^{PG}\left(t\right)\theta_{n}^{PG}+\psi_{n}\left(t\right),\text{for }n=1{,}^{\ldots },N \end{array}$$

where $\Phi_{n}^{PG}\left(t\right)$ denotes the regression vector obtained from the corresponding expression data; and $\theta_{n}^{PG}$ represents the unknown parameter vector of the pathogen gene *n* in the pathogen GRN to be estimated. The Equation (15) can be augmented for *F*_*n*_ time points of the *n*th pathogen-gene as follows,

(16) $$\begin{array}{lll} {\text{V}}_{n}^{PG} & = & \Phi_{n}^{PG}\theta_{n}^{PG}+\Gamma_{n}^{PG}\\ \text{where }{\text{V}}_{n}^{PG} & = & \left[\begin{array}{c} v_{n}\left(t_{2}\right)\\ v_{n}\left(t_{3}\right)\\ \vdots \\ v_{n}\left(t_{F_{n}}+1\right) \end{array}\right],\Phi_{n}^{PG}=\left[\begin{array}{c} \phi_{n}^{PG}\left(t_{1}\right)\\ \phi_{n}^{PG}\left(t_{2}\right)\\ \vdots \\ \phi_{n}^{PG}\left(t_{F_{n}}\right) \end{array}\right],\text{ and}\\ \Gamma_{n}^{PG} & = & \left[\begin{array}{c} \psi_{n}\left(t_{1}\right)\\ \psi_{n}\left(t_{2}\right)\\ \vdots \\ \psi_{n}\left(t_{F_{n}}\right) \end{array}\right]. \end{array}$$

The system identification method of the pathogen GRN Equation (3) can then be formulated by the following constrained least square parameter estimation problem,

(17) $$\begin{array}{l} \underset{\theta_{n}^{PG}}{\min}\frac{1}{2}{\left\|\Phi_{n}^{PG}\theta_{n}^{PG}-{\text{V}}_{n}^{PG}\right\|}_{2}^{2}\\ \text{Subject to}\overset{M_{n}}{\overset{︷}{[\begin{array}{ccc} 0 & \cdots & 0\\ \vdots & \ddots & \vdots \\ 0 & \cdots & 0\\ 0 & \cdots & 0\\ 0 & \cdots & 0 \end{array}}}\overset{K_{n}}{\overset{︷}{\begin{array}{ccc} 1 & \cdots & 0\\ \vdots & \ddots & \vdots \\ 0 & \cdots & 1\\ 0 & \cdots & 0\\ 0 & \cdots & 0 \end{array}}}\begin{array}{cc} 0 & 0\\ \vdots & \vdots \\ 0 & 0\\ 1 & 0\\ 0 & -1 \end{array}]\theta_{n}^{PG}\leq \left[\begin{array}{c} 0\\ \vdots \\ 0\\ 1\\ 0 \end{array}\right] \end{array}$$

By solving the problem in (17), we can obtain the parameters in the pathogen GRN Equation (3) and simultaneously guarantee the non-positive host-miRNA repression −*g*_*nk*_ ≤ 0, the non-positive pathogen-gene degradation −ρ_*n*_ ≤ 0, and the non-negative pathogen-gene basal level η_*n*_ ≥ 0.

As the same process in host PPIN Equation (10), the pathogen PPIN equation (4) can be rewritten in the following linear regression form,

(18) $$\begin{array}{l} w_{m}(t+1)=[\begin{array}{llll} w_{m}(t)w_{1}(t) & \cdots & w_{m}(t)w_{M_{m}}(t) & w_{m}(t)y_{1}(t) \end{array}\\ \begin{array}{lllll} \cdots & w_{m}(t)y_{J_{m}}(t) & v_{n}(t) & w_{m}(t) & 1 \end{array}]\left[\begin{array}{c} h_{m1}\\ \vdots \\ h_{mM_{m}}\\ \begin{array}{c} d_{m1}\\ \begin{array}{c} \begin{array}{c} \vdots \\ d_{mJ_{m}} \end{array}\\ \begin{array}{c} \beta_{m}\\ \begin{array}{c} 1-\sigma_{m}\\ \varepsilon_{m} \end{array} \end{array} \end{array} \end{array} \end{array}\right]+\tau_{m}(t) \end{array}$$

which could be simply represented as follows,

(19) $$\begin{array}{l} w_{m}\left(t+1\right)=\phi_{m}^{PP}\left(t\right)\theta_{m}^{PP}+\tau_{m}\left(t\right),\text{ for }m=1{,}^{\ldots },M \end{array}$$

where $\Phi_{m}^{PP}\left(t\right)$ denotes the regression vector obtained from the corresponding expression data; and $\theta_{m}^{PP}$ represents the unknown parameter vector of the pathogen protein *m* in the pathogen PPIN to be estimated. The Equation (19) can be augmented for *F*_*m*_ time points of the *m*th pathogen-protein as follows,

(20) $$\begin{array}{lll} {\text{W}}_{m}^{PP} & = & \Phi_{m}^{PP}\theta_{m}^{PP}+\Gamma_{m}^{PP}\\ \text{where }{\text{W}}_{m}^{PP} & = & \left[\begin{array}{c} w_{m}\left(t_{2}\right)\\ w_{m}\left(t_{3}\right)\\ \vdots \\ w_{m}\left(t_{F_{m}}+1\right) \end{array}\right],\Phi_{m}^{PP}=\left[\begin{array}{c} \phi_{m}^{PP}\left(t_{1}\right)\\ \phi_{m}^{PP}\left(t_{2}\right)\\ \vdots \\ \phi_{m}^{PP}\left(t_{F_{m}}\right) \end{array}\right],\text{and}\\ \Gamma_{m}^{PP} & = & \left[\begin{array}{c} \tau_{m}\left(t_{1}\right)\\ \tau_{m}\left(t_{2}\right)\\ \vdots \\ \tau_{m}\left(t_{F_{m}}\right) \end{array}\right] \end{array}$$

The system identification method of the pathogen PPIN equation (4) can then be formulated by the following constrained least square parameter estimation problem,

(21) $$\begin{array}{l} \underset{\theta_{m}^{PP}}{\min}\frac{1}{2}{\left\|\Phi_{m}^{PP}\theta_{m}^{PP}-{\text{W}}_{m}^{PP}\right\|}_{2}^{2}\\ \text{Subject to}\overset{M_{m}+J_{m}}{\overset{︷}{[\begin{array}{ccc} 0 & \cdots & 0\\ 0 & \cdots & 0\\ 0 & \cdots & 0 \end{array}}}\begin{array}{ccc} -1 & 0 & 0\\ 0 & 1 & 0\\ 0 & 0 & -1 \end{array}]\theta_{m}^{PP}\leq \left[\begin{array}{c} 0\\ 1\\ 0 \end{array}\right] \end{array}$$

By solving the problem in (21), we can obtain the parameters in the pathogen PPIN Equation (4) and simultaneously guarantee the non-negative pathogen-protein coding rate β_*m*_ ≥ 0, the non-positive pathogen-protein degradation −σ_*m*_ ≤ 0, and the non-negative pathogen-protein basal level ε_*m*_ ≥ 0. For the accuracy of the system identification method, we needed to interpolate extra time points (5 times number of the parameters, $\theta_{i}^{HG}$ in host GRN, $\theta_{j}^{HP}$ in host PPIN, $\theta_{n}^{PG}$ in pathogen GRN, and $\theta_{m}^{PP}$ in pathogen PPIN to be estimated) by using the cubic spline method to avoid the overfitting in the parameter estimation process (Johansson, 1993). The solutions of the above constrained least square parameter estimation problems in (9), (13), (17), and (21) can be obtained by using the function *lsqlin* in MATLAB optimization toolbox based on a reflective Newton method for minimizing a quadratic function (Coleman and Li, 1996). The connection weights in cross-talk GWGEIN in Mϕs and *Mtb* (or DCs and *Mtb*) during infection process can be finally solved one gene by one gene (or one protein by one protein) by using the corresponding microarray data. Since genome-wide expression measurement of protein behaviors in Mϕs, DCs and *Mtb* have not been realized yet and gene expressions are proportional to their corresponding proteins, in which 73% variance of protein abundance can be explained by mRNA abundance (Lu et al., 2007), the microarray of gene expressions can replace protein expressions for the above constrained least square parameter estimation problems in (9), (13), (17) and (21).

### System order detection scheme of the dynamic models of GWGEIN

Owing to the candidate cross-talk GWGEIN obtained from all computational and experimental predictions, we the applied a system order detection scheme to the host GRN model in (6), the host PPIN model in (11), the pathogen GRN model in (15) and the pathogen PPIN model in (19) to prune false-positives in the candidate network using the microarray data of Mϕs, DCs and *Mtb*. Based on the theory of system identification (Johansson, 1993), the insignificant parameters in the models of GWGEIN that were out of system order (association number in network) were deleted according to Akaike information criterion (AIC). Therefore, false-positives of the candidate cross-talk GWGEIN were deleted by AIC using the microarray data of Mϕs, DCs and *Mtb* and we finally obtained the real cross-talk GWGEINs between Mϕs and *Mtb* and between DCs and *Mtb* during infection process. In host GRN model (6), AIC of the *i*th host-gene could be defined by the following equation,

(22) $$\begin{array}{lll} AIC_{i}^{HG}\left(J_{i}+K_{i}\right) & = & \text{log}\left(\begin{array}{c} \frac{1}{F_{i}}\overset{t_{Fi}}{\underset{t=1}{\sum }}{\left(x_{i}\left(t+1\right)-\phi_{i}^{HG}\left(t\right){\hat{\theta }}_{i}^{HG}\right)}^{T}\\ \;\left(x_{i}\left(t+1\right)-\;\phi_{i}^{HG}\left(t\right){\hat{\theta }}_{i}^{HG}\right) \end{array}\right)\\ +\frac{2\left(J_{i}+K_{i}\right)}{F_{i}} \end{array}$$

where ${\hat{\theta }}_{j}^{HG}$ denotes the estimated parameter of the *i*th host gene by solving the problem in (9); and the estimated residual error is ${{\hat{\sigma }}_{HG,i}}^{2}$ = $\overset{tF_{i}}{\underset{t=1}{\sum }}{\left(x_{i}\left(t+1\right)-\phi_{i}^{HG}\left(t\right){\hat{\theta }}_{i}^{HG}\right)}^{T}$ $\left(x_{i}\left(t+1\right)-\phi_{i}^{HG}\left(t\right){\hat{\theta }}_{i}^{HG}\right)$ /*F*_*i*_. By the tradeoff between residual error and parameter association number, the minimum $AIC_{i}^{HG}$ in (22) can be achieved at the number $J_{i}^{*}+K_{i}^{*}$ of the real gene/miRNA regulations in host GRN (Johansson, 1993; Li and Chen, 2016; Li et al., 2016). Therefore, the real host GRN of the cross-talk GWGEIN can be solved by the minimum $AIC_{i}^{HG}$ in (22) one gene by one gene.

Similarly, in host PPIN model (11), AIC of the *j*th host-protein could be defined by the following equation,

(23) $$\begin{array}{lll} AIC_{j}^{HP}\left(J_{j}+M_{j}\right) & = & \text{log}\left(\begin{array}{c} \frac{1}{F_{j}}\overset{t_{Fj}}{\underset{t=1}{\sum }}{\left(y_{j}\left(t+1\right)-\phi_{j}^{HP}\left(t\right){\hat{\theta }}_{j}^{HP}\right)}^{T}\\ \;\left(y_{j}\left(t+1\right)-\phi_{j}^{HP}\left(t\right){\hat{\theta }}_{j}^{HP}\right) \end{array}\right)\\ +\frac{2\left(J_{j}+M_{j}\right)}{F_{j}} \end{array}$$

where ${\hat{\theta }}_{j}^{HP}$ denotes the estimated parameter of the *j*th host protein by solving the problem in (13); and the estimated residual error is ${\hat{\sigma }}_{HP,j}^{2}$ = $\overset{t_{Fj}}{\underset{t=1}{\sum }}{\left(y_{j}\left(t+1\right)-\phi_{j}^{HP}\left(t\right){\hat{\theta }}_{j}^{HP}\right)}^{T}$ $\left(y_{j}\left(t+1\right)-\phi_{j}^{HP}\left(t\right){\hat{\theta }}_{j}^{HP}\right)$/*F*_*j*_. By the tradeoff between residual error and parameter association number, the minimum $AIC_{j}^{HP}$ in (23) can be achieved at the number $J_{j}^{*}+M_{j}^{*}$ of the real PPIs in host PPIN. Therefore, the real host PPIN of the cross-talk GWGEIN can be solved by the minimum $AIC_{j}^{HP}$ in (23) one protein by one protein. Similar to the above definitions in host GRN (22) and PPIN (23), AICs of the *n*th pathogen-gene and the *m*th pathogen-protein could be respectively defined by the following equations,

(24) $$\begin{array}{lll} AIC_{n}^{PG}\left(M_{n}+K_{n}\right) & = & \text{log}\left(\begin{array}{c} \frac{1}{F_{n}}\overset{t_{Fn}}{\underset{t=1}{\sum }}{\left(v_{n}\left(t+1\right)-\phi_{n}^{PG}\left(t\right){\hat{\theta }}_{n}^{PG}\right)}^{T}\\ \;\left(v_{n}\left(t+1\right)-\phi_{n}^{PG}\left(t\right){\hat{\theta }}_{n}^{PG}\right) \end{array}\right)\\ +\frac{2\left(M_{n}+K_{n}\right)}{F_{n}} \end{array}$$

(25) $$\begin{array}{lll} AIC_{m}^{PP}\left(M_{m}+J_{m}\right) & = & \text{log}\left(\begin{array}{c} \frac{1}{F_{m}}\overset{t_{Fm}}{\underset{t=1}{\sum }}{\left(w_{m}\left(t+1\right)-\phi_{m}^{PP}\left(t\right){\hat{\theta }}_{m}^{PP}\right)}^{T}\\ \;\left(w_{m}\left(t+1\right)-\phi_{m}^{PP}\left(t\right){\hat{\theta }}_{m}^{PP}\right) \end{array}\right)\\ +\frac{2\left(M_{m}+J_{m}\right)}{F_{m}} \end{array}$$

where ${\hat{\theta }}_{n}^{PG}$ and ${\hat{\theta }}_{m}^{PP}$ denote the estimated parameters of the *n*th pathogen-gene and the *m*th pathogen-protein by solving the problem in (17) and (21), respectively; and the estimated residual errors of the *n*th pathogen-gene and the *m*th pathogen-protein are ${\hat{\sigma }}_{PG,n}2=\;{\sum_{t\;=\;1}^{t_{Fn}}\left(v_{n}\left(t+1\right)-\;\phi_{n}^{PG}(t){\hat{\theta }}_{n}^{PG}\right)}^{T}(v_{n}(t+1)-\;\phi_{n}^{PG}(t){\hat{\theta }}_{n}^{PG})/F_{n}$ and ${\hat{\sigma }}_{PP,m}2=\;\overset{t_{Fn}}{\underset{t=1}{\sum }}{\left(w_{m}(t+1)-\;\phi_{m}^{PP}(t){\hat{\theta }}_{m}^{PP}\right)}^{T}(w_{m}(t+1)-\;\phi_{m}^{PP}(t){\hat{\theta }}_{m}^{PP})/F_{m}$, respectively. By the tradeoff between residual error and parameter association number, the minimum $AIC_{n}^{PG}$ in (24) and the minimum $AIC_{m}^{PP}$ in (25) can be achieved at the numbers $M_{n}^{*}+K_{n}^{*}$ of the real gene/miRNA regulations in pathogen GRN and at the numbers $M_{m}^{*}+J_{m}^{*}$ of the real PPIs in pathogen PPIN, respectively. Therefore, the real pathogen GRN and PPIN of the cross-talk GWGEIN can be solved by the minimum $AIC_{n}^{PG}$ in (24) and the minimum $AIC_{m}^{PP}$ in (25), respectively.

By applying a system identification method and a system order detection scheme to the dynamic models of the cross-talk GWGEIN combined with the candidate cross-talk GWGEIN using the microarray data of Mϕs, DCs and *Mtb*, we can then identified the real cross-talk GWGEINs between Mϕs and *Mtb* (Figure S1) and between DCs and *Mtb* (Figure S2) during infection process. Information regarding the nodes and edges of GWGEINs are shown in Tables 1, 2, respectively. Since the host-pathogen interaction process in *Mtb* infection is very complex, it is difficult to investigate the defensive mechanisms between host and pathogen from GWGEINs in Figures S1, S2. In this situation, we applied the PNP method to the real cross-talk GWGEINs to extract the principal network structures of the real networks.

**Table 1:** **Information regarding the nodes of the GWGEINs between Mϕs and ***Mtb*** and between DCs and ***Mtb*****.

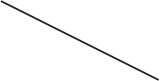	**Host-miRNAs**	**Host-TFs**	**Pathogen-TFs**	**Host-genes**	**Pathogen-genes**
Mϕs vs. *Mtb*	23	82	92	9274	3070
DCs vs. *Mtb*	21	63	87	9827	3050

**Table 2:** **Information regarding the edges of the GWGEINs between Mϕs and ***Mtb*** and between DCs and ***Mtb*****.

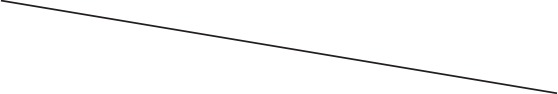	**Mϕs vs. *Mtb***	**DCs vs. *Mtb***
Host-miRNA repressions of host-genes	387	44
Host-miRNA repressions of pathogen-genes	1	0
Host-TF regulations of host-genes	554	364
Pathogen-TF regulations of pathogen-genes	3468	3476
Host-TF and pathogen-protein interactions	45	24
Pathogen-TF and host-protein interactions	1	1
Host PPIs	78,502	101,452
Pathogen PPIs	5758	5783
Host-pathogen PPIs	1086	1169

### Core network identification of the real cross-talk GWGEIN by applying the PNP method

Before using the PNP method, it is necessary to construct a combined network matrix *H* containing the estimated parameters in the real cross-talk GWGEIN as follows:

$$\begin{array}{l} H=\left[\begin{array}{cccccc} {\hat{b}}_{11} & \cdots & {\hat{b}}_{1J} & {\hat{d}}_{11} & \cdots & {\hat{d}}_{1M}\\ \vdots {\hat{b}}_{jl} & \vdots & \vdots & {\hat{d}}_{jm} & \vdots \\ {\hat{b}}_{J1} & \cdots & {\hat{b}}_{JJ} & {\hat{d}}_{J1} & \cdots & {\hat{d}}_{JM}\\ {\hat{d}}_{11} & \cdots & {\hat{d}}_{1J} & {ĥ}_{11} & \cdots & {ĥ}_{1M}\\ \vdots & {\hat{d}}_{mj} & \vdots & \vdots & {ĥ}_{mq} & \vdots \\ {\hat{d}}_{M1} & \cdots & {\hat{d}}_{MJ} & {ĥ}_{M1} & \cdots & {ĥ}_{MM}\\ {â}_{11} & \cdots & {â}_{1J} & 0 & \cdots & 0\\ \vdots & {â}_{ij} & \vdots & \vdots & \ddots & \vdots \\ {â}_{J1} & \cdots & {â}_{JJ} & 0 & \cdots & 0\\ 0 & \cdots & 0 & {ê}_{11} & \cdots & {ê}_{1M}\\ \vdots & \ddots & \vdots & \vdots & {ê}_{nm} & \vdots \\ 0 & \cdots & 0 & {ê}_{M1} & \cdots & {ê}_{MM}\\ -{ĉ}_{11} & \cdots & -{ĉ}_{J1} & -{ĝ}_{11} & \cdots & -{ĝ}_{M1}\\ \vdots & -{ĉ}_{ik} & \vdots & \vdots & -{ĝ}_{nk} & \vdots \\ -{ĉ}_{1K} & \cdots & -{ĉ}_{JK} & -{ĝ}_{1K} & \cdots & -{ĝ}_{MK} \end{array}\right]\\ \in {\mathbb{R}}^{\left(2J\;+\;2M\;+\;K\right)\;\times \;\left(J\;+\;M\right)} \end{array}$$

where â_*ij*_ and −ĉ_*ik*_ were obtained in ${\hat{\theta }}_{i}^{HG}$ estimated in (9) and (22); ${\hat{b}}_{jl}$ and ${\hat{d}}_{jm}$ were obtained in ${\hat{\theta }}_{j}^{HP}$ estimated in (13) and (23); ê_*nm*_ and −ĝ_*nk*_ were obtained in ${\hat{\theta }}_{n}^{PG}$ estimated in (17) and (24); and ĥ_*mq*_ and ${\hat{d}}_{mj}$ were obtained in ${\hat{\theta }}_{m}^{PP}$ estimated in (21) and (25). â_*ij*_ and ê_*nm*_ denote transcriptional regulatory abilities in the intraspecies GRNs of host and pathogen, respectively; ${\hat{b}}_{jl}$ and ĥ_*mq*_ indicate interactive abilities in the intraspecies PPINs of host and pathogen, respectively; −ĉ_*ik*_ and −ĝ_*nk*_ represent miRNA repression abilities in the intraspecies GRNs of host and pathogen, respectively; and ${\hat{d}}_{jm}$ and ${\hat{d}}_{mj}$ signify the interactive abilities between the *j*th host-protein and the *m*th pathogen-protein in the interspecies PPIN between host and pathogen. The estimated weights of the network connections in intraspecies GRNs, intraspecies PPINs, and the interspecies PPIN therefore consist the network matrix *H* of the real cross-talk GWGEIN. If a connection does not exist in the candidate cross-talk GWGEIN or has been pruned by AIC, the corresponding parameter in network matrix *H* is zero. PNP is then applied to *H* to identify the core network of the real cross-talk GWGEIN. PNP is based on singular value decomposition of *H* as follows,

(26) $$\begin{array}{l} H=UDV^{T} \end{array}$$

where *U*ϵℝ^(2*J*+2*M*+*K*) × (*J*+*M*)^; *V*ϵℝ^(*J*+*M*) × (*J*+*M*)^; and *D* = **diag**(*d*_1_, …, *d*_*s*_, …, *d*_*J*+*M*_) consists of the *J*+*M* singular values of *H* in descending order, i.e., *d*_1_≥…≥*d*_*s*_≥…≥ *d*_*J*+*M*_. Note that, the **diag**(*d*_1_, *d*_2_) denotes the diagonal matrix of *d*_1_ and *d*_2_. The eigenexpression fraction (*E*_*s*_) is defined as follows,

(27) $$\begin{array}{l} E_{s}=\frac{d_{s}^{2}}{\overset{J+M}{\underset{s=1}{\sum }}d_{s}^{2}} \end{array}$$

We selected the top *Q* singular vectors of *H* with the minimal *Q* such that $\overset{Q}{\underset{s=1}{\sum }}E_{s}\geq 0.85$ which means that the *Q* principal components contain 85% core network structure of GWGEIN from the perspective of energy. The projection (*T*) of each row in *H* to the top *Q* singular vectors *V* is defined as follows,

(28) $$\begin{array}{lll} T\left(p,s\right) & = & h_{p}\times v_{s},\text{ for }p=1,\ldots ,\left(2J+2M+K\right)\\ \text{and }s=1,\ldots ,Q \end{array}$$

where *h*_*p*_ and *v*_*s*_ denote the *p*th row vector of *H* and the *s*th column vector of *V*, respectively. We then defined the 2-norm projection value of each node, i.e., gene/miRNA/protein, in the real cross-talk GWGEIN to the core network structure consisted of the top *Q* singular vectors as follows:

(29) $$\begin{array}{l} D\left(p\right)={\left[\sum_{s=1}^{Q}T{\left(p,s\right)}^{2}\right]}^{1/2},\text{ for }p=1,\ldots ,\left(2J+2M+K\right) \end{array}$$

If *D*(*p*) is close to zero, the *p*th node is almost independent of the core network structure consisted of the top *Q* singular vectors. Because the purpose of the identification of the core network is to investigate the offensive and defensive mechanisms between host and pathogen from a perspective of signal transduction pathways, the proteins with top *D*(*p*) from receptors to TFs and their connected miRNAs and genes are chosen as the core proteins/genes/miRNAs to consist the core network. Finally, we extracted the HPCNs from the real cross-talk GWEGINs between Mϕs vs. *Mtb* and DCs vs. *Mtb* (Figures S3, S4), respectively. Accordingly, HPCNs possess the principal structure that represents the core GWGEINs during *Mtb* infection.

## Results and discussion

### GWGEINS of Mϕs and DCs infected with *Mtb*

**Figure 2:**
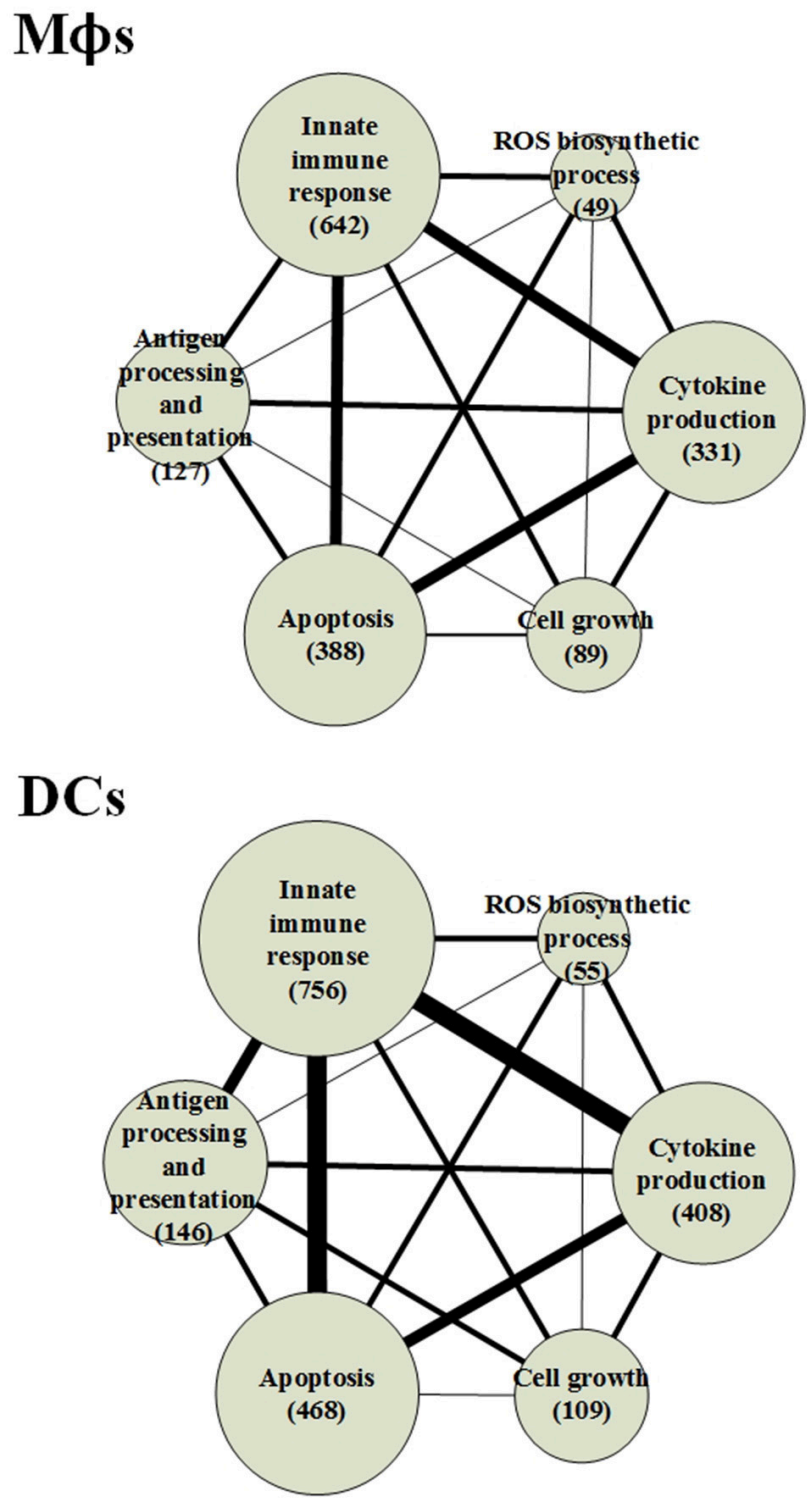
**The functional networks of GWGEINs in Mϕs and DCs during early ***Mtb*** infection**. After constructing the GWGEINs of Mϕs and DCs during *Mtb* infection, we used gene ontology (GO) to analyze the biological processes in both cell types. The number below each function represents the gene number in GWGEINs that participate in the function. By comparing the gene number of each function between Mϕs and DCs, we found that DCs are able to more effectively control *Mtb* infection than Mϕs.

The GWGEINs of Mϕs and DCs are shown in Figures S1, S2, respectively. The number of nodes and edges are shown in Tables 1, 2, respectively. There was no significant difference in the number of nodes between Mϕs and DCs. However, the edges between both cell types in Table 2 demonstrated remarkable difference in host-miRNAs to host-genes (DC:44, Mϕ:387) and host PPIs (DC:101452, Mϕ:78502). The results show that there are more miRNA regulations in Mϕs than in DCs. Since more genes are inhibited by miRNAs, it could result in that there are less PPIs in Mϕs than in DCs. Interestingly, the identified PPIs between pathogen TFs and host proteins (i.e., Rv1423 negatively interacts with BNIP3L, −16 in Mϕs and −27 in DCs) present in both cell types indicate that Rv1423 may interfere with BNIP3L, which is involved in the apoptosis in Mϕs and DCs. We also plotted the functional networks of GWGEINs in Mϕs and DCs (Figure 2). The number of genes participating in the innate immune response, antigen processing and presentation, cytokine production, and apoptosis was much higher in DCs than in Mϕs, suggesting that DCs are more responsive to the infection of *Mtb* than Mϕs. However, the GWGEINs did not contain sufficient information for us to investigate the defensive mechanisms between host and pathogen. Thus, we extracted HPCNs from GWGEINs in both Mϕs and DCs during early *Mtb* infection via the PNP method (Figures S3, S4).

### HPCNs in Mϕs and DCs infected with *Mtb*

#### The biological processes of the host core networks in both cell types

**Figure 3:**
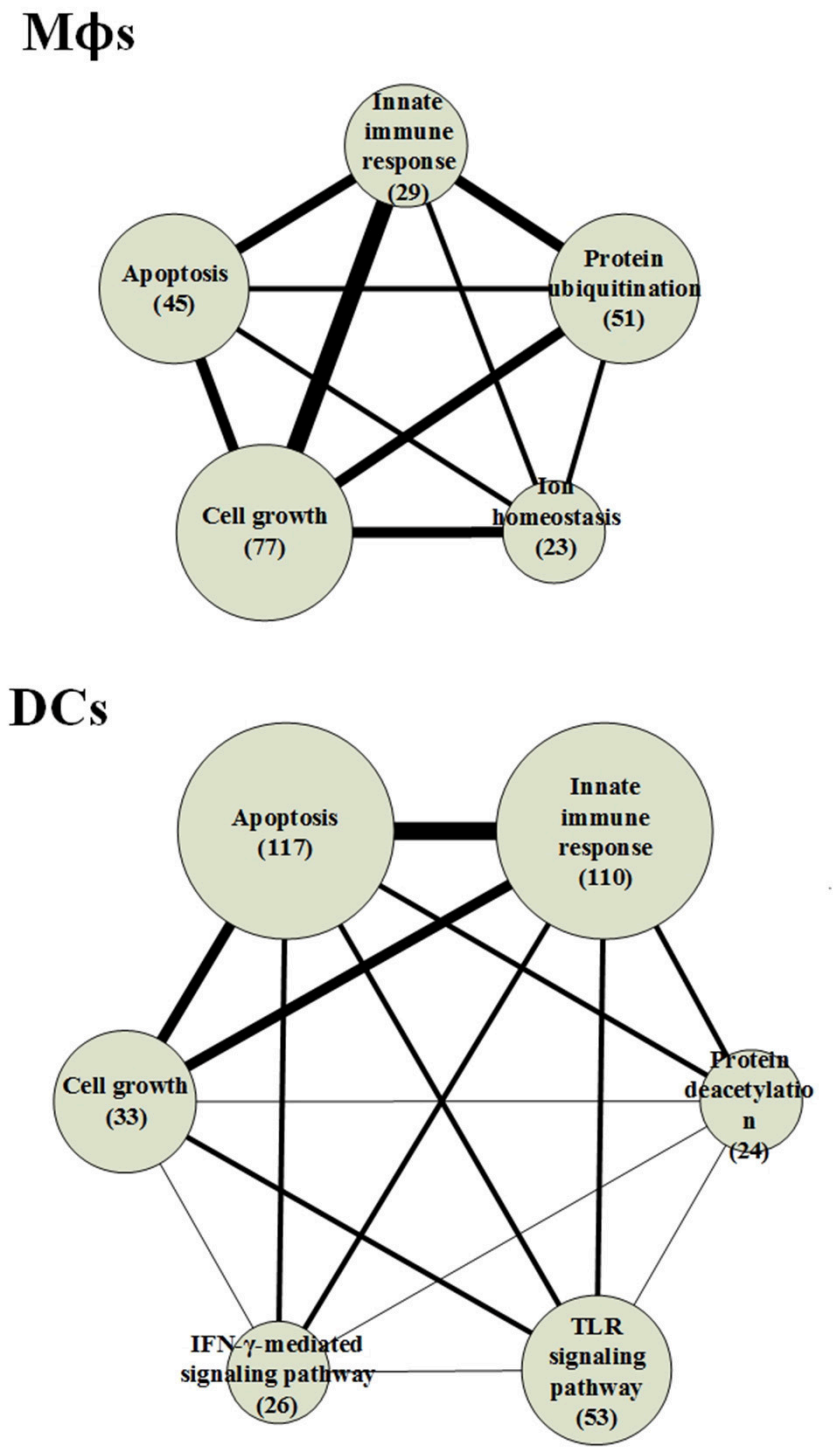
**The functional networks of HPCNs in Mϕs and DCs during early ***Mtb*** infection**. We used gene ontology (GO) to analyze the biological processes of host-pathogen core networks (HPCNs) in Mϕs and DCs during *Mtb* infection. The gene number participating in cell growth in Mϕs is higher than in DCs, whereas the gene number participating in apoptosis in DCs is higher than Mϕs. Thus, DCs tend to undergo apoptosis, and can avoid the influence from *Mtb*, whereas Mϕs tend to go into cell growth and can be easily influenced by *Mtb*. Protein ubiquitination and ion homeostasis are only present in Mϕs, which are required for Mϕs to adapt to the rapid changes during infection. The IFN-γ-mediated and toll-like receptor (TLR) signaling pathways are only present in DCs. In addition, protein deacetylation in DCs may protect DCs from the *Mtb* infectious mechanisms.

By using the PNP method, we extracted the HPCNs in Mϕs and DCs infected with *Mtb* as shown in Figures S3, S4, respectively. In order to get an overview of the molecular mechanisms of the host during infection, we used gene ontology (GO) to analyze the biological processes of the host core networks in Mϕs and DCs. Furthermore, based on the PPIs of HPCNs, we constructed the functional networks of HPCNs in Mϕs and DCs as shown in Figure 3.

The result indicates that the number of genes participating in the innate immune response in DCs was higher than in Mϕs and the 110 innate immune response genes in DCs were transcriptionally affected by 700 positive regulations and 653 negative regulations. In order to adapt to the infection of *Mtb*, post-translation epigenetic modifications can be found in HPCNs such as ubiquitination in Mϕs and deacetylation in DCs. These epigenetic modifications can be detected by the basal level of κ_*j*_ in the host protein expression model in (2). The host proteins (IRF2 and SUMO1) in IFN-γ-mediated pathway, regulated by the deacetylase protein (HDAC1), the SUMO proteins (SUMO1, SUMO2, and SUMO3), and the ubiquitin proteins (UBC and MUL1), and the host protein (UBC) in TLR signaling pathway, regulated by the deacetylase protein (HDAC1), the acetyltransferase protein (DLAT), the methyltransferase-associated protein (MTAP), the SUMO proteins (SUMO1, SUMO2, and SUMO3), and the ubiquitin proteins (UBC and MUL1), present in DCs can activate the immune response. In addition, the host proteins (AP3D1, ATG5, and PTPRC) in ion homeostasis, regulated by the ubiquitin protein (UBC) and the SUMO protein (SUMO2), are induced in Mϕs to counteract the increase in metal ions during infection. Mϕs can induce apoptosis during infection but *Mtb* can block this process. The number of cell growth-related genes was higher than those related to apoptosis (Figure 3), which facilitates the survival of *Mtb* in Mϕs. However, in DCs, there were more apoptotic genes than cell growth genes. Additionally, in Mϕs, two pro-apoptotic proteins (MOAP1, and APAF1) were involved in apoptosis, while, in DCs, three pro-apoptotic proteins (BCL2, CFLAR, and AIFM1) and one anti-apoptotic protein (XIAP) were involved in apoptosis. We further identified that the host protein SKP1, involved in the innate immune response, positively regulates the pro-apoptotic proteins in DCs. This indicates that DCs are less susceptible than Mϕs and readily induce apoptosis mediated by the innate immune response as a means of eliminating *Mtb*.

#### Host-pathogen cross-talk interactions in both cell types

**Figure 4:**
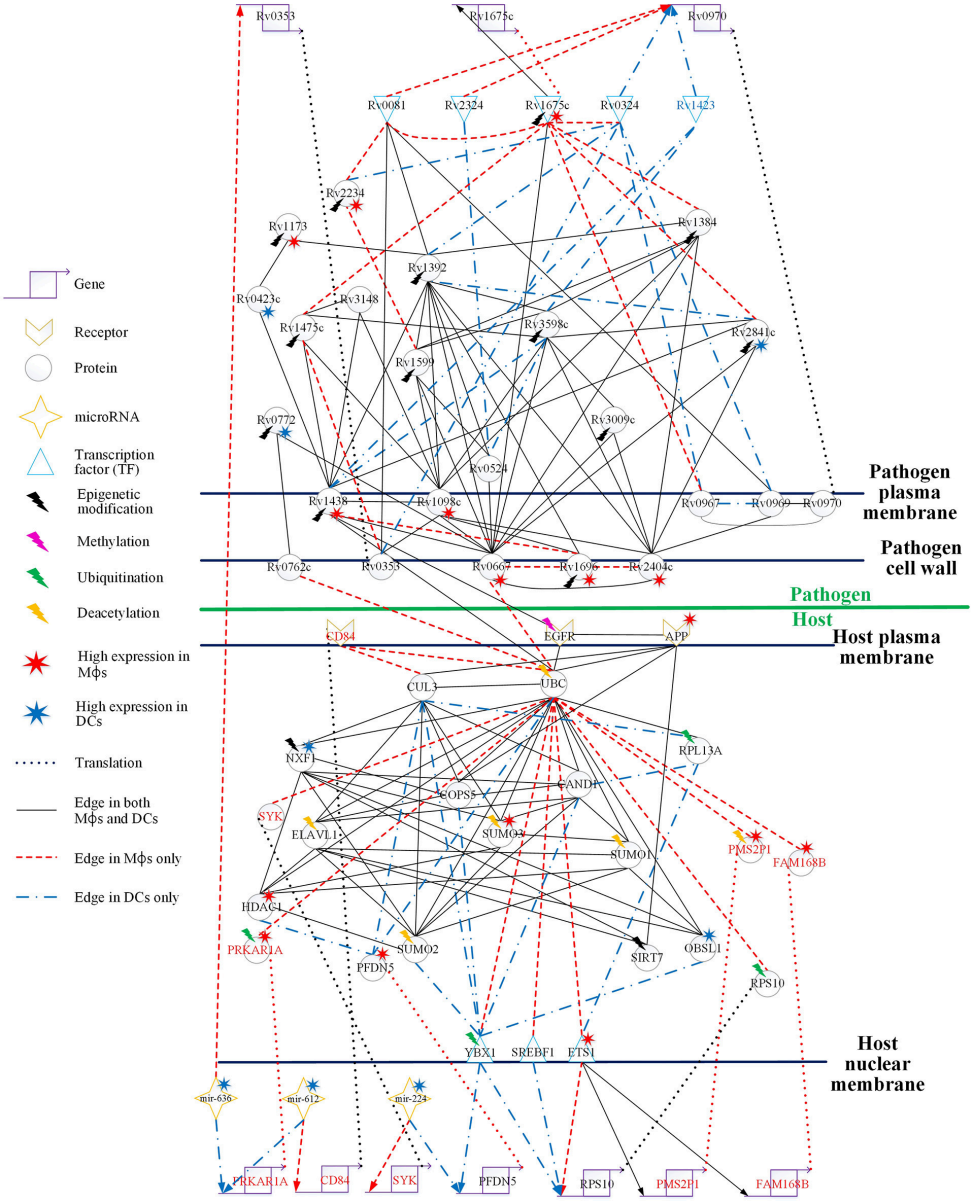
**Comparison of the HPCNs in Mϕs and DCs infected with ***Mtb*** during early infection**. Host-pathogen core networks (HPCNs) contain the major structure of GWGEINs via the principal network projection (PNP) method, which allowed investigation of the underlying mechanisms of the host and pathogen. HPCNs highlight the different signaling pathways between Mϕs and DCs during *Mtb* infection. The upper half shows the pathogen network and the lower half the host network. The edges with solid black lines represent presence in both cell types. The names in red, blue, and black represent the proteins specific in Mϕs, DCs, and both cell types, respectively. We denote the lightning bolts to represent proteins with epigenetic modifications due to the significant basal level changes between Mϕs and DCs.

The cross-talk between pathogen and host has been widely investigated. However, the real genetic and epigenetic network connections and host-pathogen PPIs are still unclear. Here, we compared HPCNs to show the common and different cross-talk interactions between Mϕs and DCs during *Mtb* infection (Figure 4). It has shown that 126 *Mtb* genes found by the analysis of transposon mutant pools to be required for survival are constitutively expressed rather than regulated at least in primary mouse Mϕs (Rengarajan et al., 2005). Seven *Mtb* proteins (Rv1049, Rv1681, Rv1337, Rv3868, Rv0928, Rv3283, and Rv3269) of the HPCN in Mϕs infected with *Mtb* (Figure S3) and three *Mtb* proteins (Rv1049, Rv0082, and Rv3369) of the HPCN in DCs infected with *Mtb* (Figure S4), encoded by the *Mtb* genes required for the survival of *Mtb* in host, have been identified in this study. Additionally, by identifying mutations that alter the phenotypic consequence (i.e., genetic interactions) of inactivating a gene of interest, 66 *Mtb* genes that encode the proteins which associate to form multisubunit transporters required for *Mtb* survival in the host have also been found (Joshi et al., 2006). Two *Mtb* proteins (Rv2004c, and Rv3877) of the HPCN in Mϕs infected with *Mtb* (Figure S3) and an *Mtb* protein (Rv0427c) of the HPCN in DCs infected with *Mtb* (Figure S4), encoded by the *Mtb* genes involved in transporter assembly required for *Mtb* survival in host, have been also identified in this study. However, the comparison between two HPCNs in Mϕs and DCs infected with *Mtb* (Figure 4) does not contain any essential *Mtb* gene/protein required for the survival of *Mtb* in host. The comparison result in Figure 4 comprises 11 differentially expressed pathogen proteins and 17 non-differentially expressed pathogen proteins between two host cells. It is because the result in Figure 4 contains the proteins or genes, which have the most differential interactions or regulations between two HPCNs in Mϕs and DCs infected with *Mtb*. It intuitively indicates that the *Mtb* essential genes/proteins were involved in the HPCNs in Mϕs and DCs infected with *Mtb* to respectively assist the different mechanisms between Mϕs and DCs infected with *Mtb* from a perspective of signal transduction pathway.

In Figure 4, there are three pathogen proteins (Rv0667, Rv0762c, and Rv1438) interacting with host proteins (UBC, EGFR). In addition, there are other pathogen proteins (Rv2404c, Rv1696, and Rv1098c) that may help Rv0667, Rv0762c, and Rv1438 promote host-pathogen interactions. This suggests that Mϕs are more easily influenced by *Mtb* than DCs during early infection, and therefore specific interactions are present only in Mϕs. Moreover, Rv2404c, Rv1696, and Rv1098c (their expression was significantly activated in Mϕs (*p* < 0.05) rather than in DCs) promote cross-talk interactions in Mϕs, inducing the influence on Mϕ invasion. In addition, Rv2404c, Rv0667, Rv0762c, Rv1098c, Rv1696, and Rv1438 are localized on the cell wall. This is consistent with the pathogenesis of *Mtb* in which membrane proteins influence the host immune response and enhance pathogen survival ability. The host protein UBC associates with the toll-like receptor-signaling pathway. We identified that the host protein UBC is negatively affected by the pathogen via Rv0667, Rv0762c, and Rv1438 Mϕs, while it is positively affected by the pathogen Rv1438 in DCs. Therefore, *Mtb* can alter the host immune system in both Mϕs and DCs by binding to UBC. In addition, UBC plays a role in phagocytosis as well as antigen processing and presentation processing (Peters et al., 1998). Accordingly, *Mtb* avoids the host antigen processing and presentation processing by binding to UBC in both Mϕs and DCs. Although, *Mtb* affects UBC in both cell types, the three negative interactions of UBC by pathogen in Mϕs result in more influence on the immune system and antigen presentation than in DCs. This accounts for why Mϕs are more susceptible to *Mtb* than DCs.

EGFR (epidermal growth factor receptor) is involved in cell growth. Pathogen protein Rv1438 interacts with EGFR in both Mϕs and DCs, and affects the process of cell growth (Figure 4). By doing this, *Mtb* can survive in host cells without being killed by the apoptosis process. Because *Mtb* promotes cell growth, a recent study has shown that the inhibition of EGFR could restrict the growth of *Mtb* (Stanley et al., 2014). Furthermore, EGFR is also involved in the MAPK signaling pathway, which transducts extracellular signals from receptors on the membrane to the DNA in the nucleus of the cell. We identified that the oxidative stress responsive proteins (OXSR1, and OSGIN1) are negatively affected by UBC in Mϕs. Moreover, it has been proposed that *Helicobacter pylori* infection induces oxidative stress (Ding et al., 2007), which could increase the DNA mutation risk by inhibiting oxidative stress responsive proteins (McAdam et al., 2016). The interaction of the pathogen protein, Rv1438, may cause the dysregulated EGFR in Mϕs and influence the MAPK signaling pathway. Although Rv1438 interacts with EGFR during *Mtb* infection of Mϕs and DCs, the expression of Rv1438 is higher in *Mtb* and the oxidative stress responsive proteins (OXSR1, and OSGIN1) are negatively affected by UBC, infecting Mϕs, and causing more reactive intermediates that could lead to mutations in macrophages during infection in Mϕs during *Mtb* infection. EGFR is not only associated with cell growth but is also involved in the PI3K-Akt signaling pathway, which plays a role in the signal transduction pathways of cytokines. Rv1438 can interrupt the immune response, causing a delay in inflammation, thus expediting the invasion of *Mtb* during early infection. In addition, it has been reported that Rv1438 is an essential gene for *in vitro* growth of *Mtb* H37Rv (Griffin et al., 2011). Taken together, Rv1438 can induce EGFR dysregulation through the interspecies interaction between them, which interrupts antigen presentation via UBC. Due to its participation in several cellular functions in the host, Rv1438 could be a potential drug target for future treatment of tuberculosis.

#### Host responses in Mϕs and DCs during Mtb infection

Human cells possess the ability to change the behavior of proteins in order to adapt to rapidly changing circumstance. This is referred to as epigenetics or post-translational modifications, which include methylation, sumoylation, ubiquitination, and acetylation. The epigenetic influence on proteins is more efficient than the genetic influence on DNA transcription for adaptation to the changing environment during infection.

In both cell types, there are proteins implicated in ubiquitination such as UBC and CUL3, deacetylation such as HDAC1 and SIRT7, and sumoylation such as SUMO1, SUMO2, and SUMO3 (Figure 4). Ubiquitination can affect proteins in many ways such as inducing degradation via the proteasome, and altering their cellular location. As shown in Figure 4, UBC has edges that are more specific in Mϕs than they are in DCs, and CUL3 participates in DCs more than in Mϕs. In addition, the expression of HDAC1 is higher in DCs, and there are differential basal levels of UBC between Mϕs and DCs. This indicates that HDAC1 has higher activity in Mϕs and may affect UBC, which is involved in several immune responses through deacetylation, which results in a change in basal levels. Furthermore, it has been reported that HDAC inhibitors induce inhibition of the host immune response against microbial pathogens in Mϕs and DCs (Roger et al., 2011). Specifically, HDAC functions such as histone deacetylation enhance the immune response in Mϕs against bacterial infection. UBC may be affected by not only deacetylation but also by sumoylation. In addition, the proteins ELAVL1 and SUMO1/2/3 may be influenced by deacetylation via their interaction with HDAC1.

**Figure 5:**
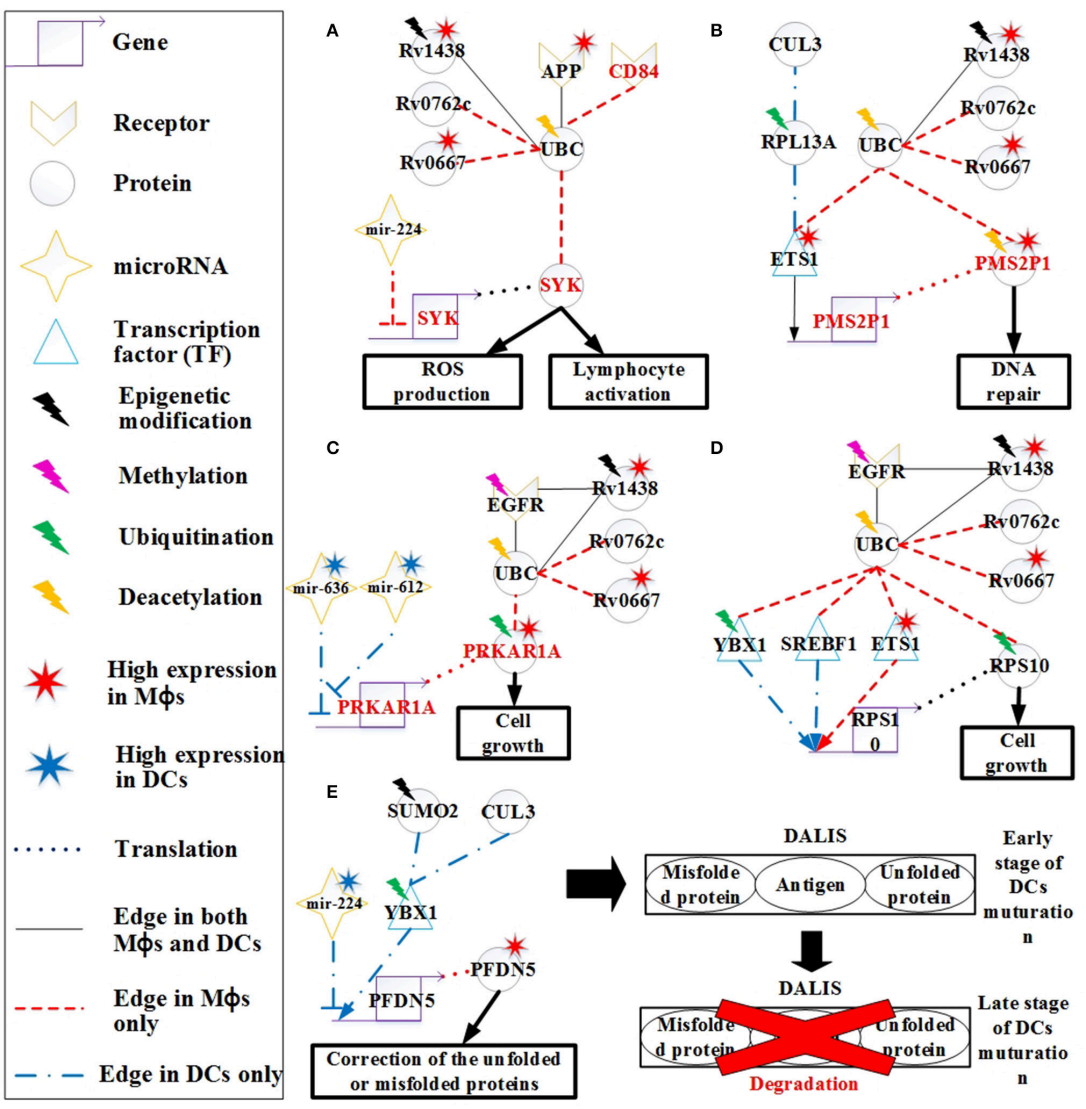
**The signaling pathways of the host molecular mechanisms based on HPCNs in Figure 4 in Mϕs and DCs during ***Mtb*** infection**. **(A)** Reactive oxygen species (ROS) production is present only in Mϕs and may be influenced by *Mtb* proteins through interaction with UBC. In addition, CD84 signals to SYK to activate lymphocytes through UBC. Deacetylation of UBC avoids the influence from *Mtb* proteins. **(B)** The high activity of DNA repair in Mϕs reflects DNA damage caused by oxidative stress in Mϕs, and is dysregulated by *Mtb* proteins in spite of the deacetylation of PMS2P1. **(C)** The induction of cell growth in Mϕs may be influenced by *Mtb* proteins through interaction with UBC despite ubiquitination of PRKAR1A and EGFR methylation. DCs avoid cell growth through the inhibition of mir-612 and mir-636. **(D)** Differential regulation of RPS10, which is involved in cell growth in both cell types, may be influenced by *Mtb* proteins through interaction with UBC. Although, RPS10 induces ubiquitination in Mϕs, *Mtb* proteins can influence RPS10 by inducing its activity in both cell types. **(E)** High expression of PFDN5 in Mϕs indicates that Mϕs tend to correct unfolded or misfolded proteins, whereas PFDN5 is inhibited by mir-224 in DCs. The accumulation of unfolded or misfolded proteins and antigens in DCs leads to formation of the dendritic cell aggresome-like induced structures (DALIS) to delay degradation via the proteasome at an early stage of maturation, until during the late stage maturation of DCs.

Oxidative stress is a reflection of the imbalance between reactive oxygen species (ROS) and the biological system's ability to detoxify the reactive intermediates or to repair the resulting damages. High ROS production and disturbances in normal redox state lead to oxidative stress. Oxidative stress is a primary response of the immune system, and the induction of ROS during infection helps the immune system to kill pathogens. However, the simultaneous production of ROS and free radicals damages cellular components, including proteins, lipids, and DNA. Amyloid precursor protein (APP) is involved in the response to oxidative stress, and the APP receptor receives oxidative stress signals induced by the immune system during *Mtb* infection. The higher APP expression in Mϕs than in DCs demonstrates that there is more oxidative stress in Mϕs. The high ROS production not only helps the host kill the pathogen but also causes DNA damage, and therefore the host cell must typically inhibit ROS production. The tyrosine kinase SYK also plays a role in ROS production (Romero et al., 2014). High expression of APP in Mϕs reflects the high oxidative stress (Figure 5A). Accordingly, APP can signal via SYK to induce ROS production. mir-224 has been characterized as an inhibitor of ROS production through silencing SYK, but the low expression of mir-224 in Mϕs demonstrates that Mϕs still need SYK to produce ROS. Inhibition of the over production of ROS is only observed in Mϕs, emphasizing the difference in defense mechanisms between Mϕs and DCs. Specifically, Mϕs prioritize killing the pathogen, whereas DCs prioritize antigen presentation to activate an adaptive immune response. The deacetylation of UBC may enhance the downstream immune response to protect the host from the invasion of *Mtb*. However, pathogen proteins Rv0667, Rv0762c, and Rv1438 may interfere with the production of SYK through interacting with UBC to prevent ROS mediated elimination of the pathogen.

CD84, a member of the signaling lymphocyte activation molecule (SLAM) family modulates several immune responses, including T cell/B cell activation and antibody production (Figure 5A). CD84 is a hemophilic receptor expressed on T cells, B cells, DCs, monocytes, and Mϕs. The expression of CD84 increases the activation of T cells, B cells, and DCs. SYK is also involved in the adaptive immune response. CD84 affects SYK through interacting with UBC in Mϕs, thus modulating the adaptive immune response. This is a mechanism employed by Mϕs to activate the adaptive immune response during infection. However, activation of adaptive immunity can be blocked by the pathogen via the interaction of UBC to prevent antibody production and promote its survival.

Other differences in the response to oxidative stress initiated in Mϕs and DCs were also identified. RPL13A plays a role in ubiquitination and assists ETS1 in its translocation to the nucleus for transcription (Figure 5B). The *PMS2P1* gene is regulated by ETS1 in Mϕs and DCs. The high expression of PMS2P1 in Mϕs could be due to the high activity of ETS1. The main function of PMS2P1 is to repair DNA damage caused by oxidative stress. Therefore, the high expression of PMS2P1 indicates that there is more oxidative stress in Mϕs, which is required for DNA repair. *Mtb* proteins could also account for the high expression of PMS2P1 in Mϕs. PMS2P1 can induce deacetylation to control its activity (Edwards et al., 2009); however, *Mtb* proteins can counteract this process, causing high expression of PMS2P1. High DNA repair activity is an important mechanism of cancer progression. In addition, ETS1 can function as an oncogene and drive tumorigenesis (Kathuria et al., 2011). The high expression of ETS1 and PMS2P1 as well as the influence from *Mtb* proteins may play a role in the progression from TB to lung cancer in Mϕs.

PRKAR1A encodes the type 1α regulatory subunit (RIα) of cAMP-dependent protein kinase (PKA). The RIα protein is upregulated in many cancer cell lines, suggesting its potential role in cell cycle regulation and growth. It has been shown that the overexpression of RIα in lung cancer (Young et al., 1995) is indicative of growth of lung cancer cells. EGFR methylation negatively regulates the EGFR downstream pathway (Montero et al., 2006). However, *Mtb* proteins can affect PRKAR1A function by interacting with EGFR and UBC despite of the methylation of EGFR and ubiquitination of PRKAR1A (Figure 5C; Gu et al., 2015). The significant change (*p* < 5.67 × 10^−14^) in the gene expression profiles of EGFR between *Mtb*-infected Mϕs and DCs supports this finding. PRKAR1A has higher expression in Mϕs than in DCs, which may cause cell growth in Mϕs. Cell growth benefits the survival of pathogen. Pathogen proteins such as Rv0667, Rv1438, and Rv0762c may interfere with the activity of PRKAR1A by interacting with UBC. The higher expression in Mϕs than DCs results from the inhibition of mir-612. The high expression of mir-612 and mir-636 in DCs inhibits PRKAR1A expression, whereas PRKAR1A is highly expressed in Mϕs due to low mir-612 and mir-636 expression in this cell type. This suggests that DCs are able to avoid dysregulation of cell growth caused by the pathogen, which indirectly reduces the ability of *Mtb* to residue in DCs.

Ribosomal protein S10 (RPS10) is also associated with cell growth, as it plays a critical role in ribosome biogenesis (Ren et al., 2010). RPS10 is regulated by TFs (YBX1, SREBF1, and ETS1) in both Mϕs and DCs (Figure 5D). Ubiquitination of RPS10 interferes with its activity, which is associated with cell growth in Mϕs (Buckley et al., 2012). However, the function of RPS10 can indirectly be blocked by *Mtb* proteins through interaction with UBC, as a means of controlling cell growth in Mϕs.

Ubiquitination of YBX1 facilitates its transport from the cytosol to the nucleus for transcriptional regulation. In Figure 5E, PFDN5 is regulated by YBX1 and mir-224, but mir-224 is expressed at higher levels in DCs than it is in Mϕs, causing higher expression of PFDN5 in Mϕs. The decreased expression of PFDN5, which plays a role in protein folding, and the oxidative stress in DCs could result in the induction of misfolded proteins. For the maintenance of cellular homeostasis, misfolded proteins must be ubiquitinated for degradation via the ubiquitin-proteasome pathway. It has been reported that ubiquitinated misfolded proteins can accumulate in DCs to form dendritic cell aggresome-like induced structures (DALIS), and the ubiquitinated proteins in DALIS are protected from degradation via the proteasome during the early stages of DC maturation (Lelouard et al., 2002). At the late stage of maturation, the proteasome actively participates in the removal of DALIS (Lelouard et al., 2002). In addition, DALIS act as an Ag (antigen of *Mtb*) storage center during DC maturation to prioritize degradation of proteins in response to infection (Canadien et al., 2005). Taken together, the formation of DALIS helps DCs promote antigen processing during DC maturation for presentation of the peptides on MHC molecules to B cells.

#### The protective mechanisms of Mtb in Mϕs and DCs

**Figure 6:**
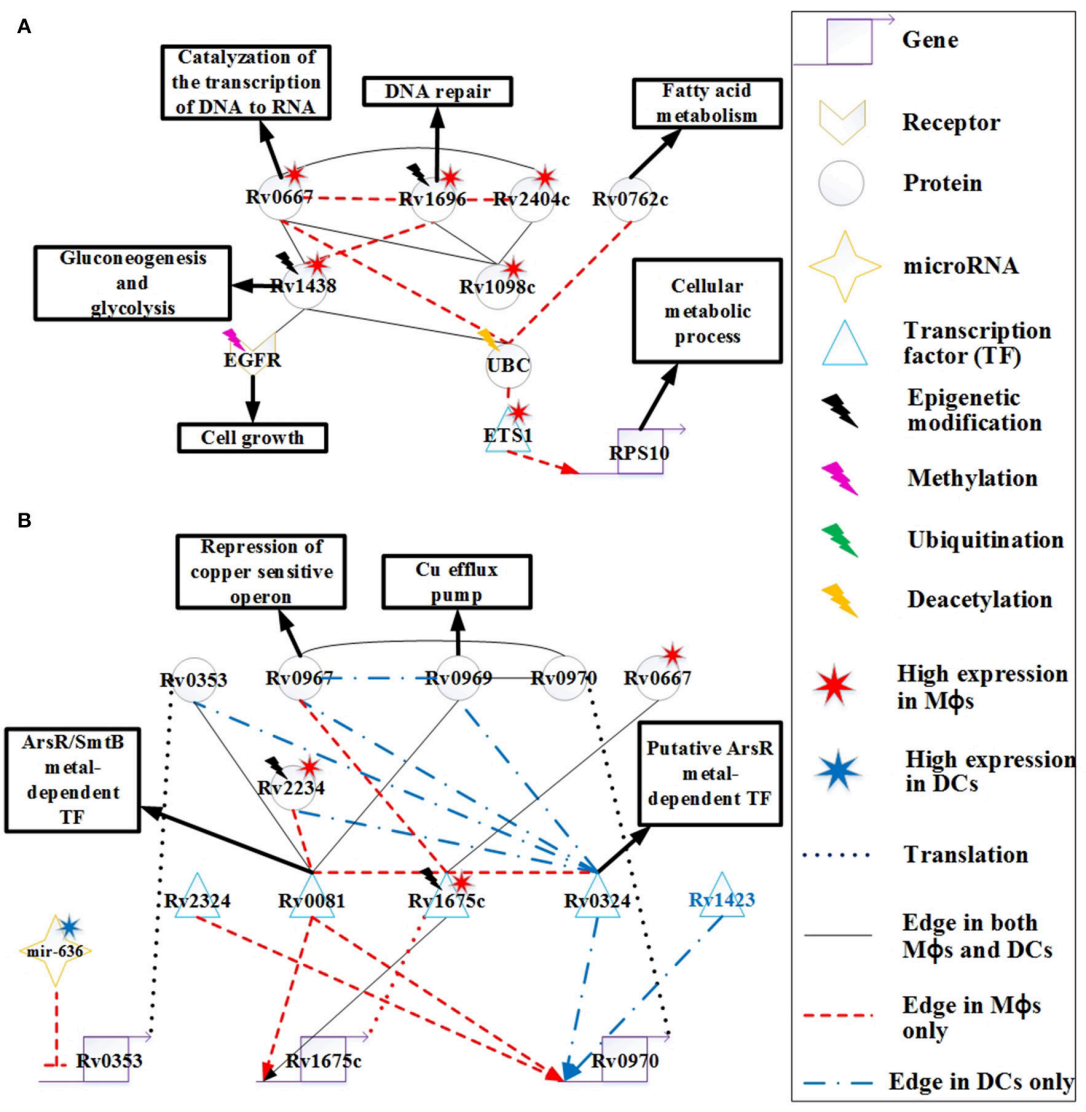
**The signaling pathways of pathogen protective mechanisms based on HPCNs in Figure 4 in Mϕs and DCs during ***Mtb*** infection**. **(A)** The proteins Rv0667, Rv1696, Rv2404c, Rv0762c, Rv1438, and Rv1098c are expressed on the cell membrane of *Mtb*, and Rv0667 and Rv1438 are essential for the survival of *Mtb*. These proteins may counteract the defensive mechanisms of both cell types through interactions with EGFR and UBC. Therefore, these proteins with higher expression and more specific edges in Mϕs than DCs could cause the dysfunction of cell growth and DNA repair in Mϕs. Some of these proteins have differential basal levels, whereas only Rv1438 has been found to undergo acetylation, which could facilitate its interaction with host proteins. **(B)** The metal-dependent homeostasis signaling pathway plays an important protective role to counteract the defense mechanisms of host cells. However, the metal-dependent homeostasis signaling pathway of *Mtb* varies between Mϕs and DCs. *Mtb* evolves this pathway to counteract the metal burst from host cells and induce metal homeostasis in Mϕs and DCs during infection. The function of Rv0970 is still unknown, whereas *rv0970* is regulated by different transcription factors (TFs) of *Mtb* in Mϕs and DCs, and interacts with Rv0967 and Rv0969 after translation, a member of the metal-dependent homeostasis signaling pathway. Rv1675c regulates itself and interacts with metal-dependent proteins or TFs in Mϕs. Furthermore, Rv1675c is highly expressed and demonstrates specific edges in Mϕs, which plays an important role in the metal-dependent homeostasis signaling pathway in Mϕs. The inhibition of mir-636 on *rv0353* in Mϕs could decrease antibody production.

Rv0667 (RpoB), a DNA-directed RNA polymerase that catalyzes the transcription of DNA to RNA, plays an important role in *Mtb* infection. There are many PPIs of *Mtb* that interact with Rv0667 in *Mtb*, infecting Mϕs and DCs (Figure 4). Rv0667 is highly expressed in *Mtb*, infecting Mϕs, and it interferes with the function of the host protein UBC to facilitate *Mtb* invasion (Figure 6A). In addition, high expression of Rv1438, Rv1098c, and Rv2404c increases the activity of Rv0667 in Mϕs. Because of its role in *Mtb* infection, RpoB is the target of the drug rifampicin and also plays a role during infection. Mutations are spontaneous and then selected under drug selection pressure. Possibly, DNA damage could increase the mutation rate overall.

The function of Rv0762c is currently unknown. It has been suggested that Rv0762c functions in fatty acid metabolism as an additional bacterial adaptation for *Mtb* to survive novel host-derived pressures within the phagosomal environment (Rohde et al., 2012). As shown in Figure 6A, Rv0762c may influence the host gene RPS10, which is involved in the cellular metabolic process via its interaction with UBC and ETS1 in Mϕs. Another metabolic enzyme, Rv1438 (TpiA), triosephosphate isomerase, is an essential enzyme for gluconeogenesis and glycolysis, and is essential for the survival of *Mtb* (Connor et al., 2011). The varying basal expression levels of Rv1438 may result from acetylation (Kuhn et al., 2014), which can enhance its interaction with host proteins. For example, Rv1438 interacts with EGFR and UBC in Mϕs and DCs (Figure 6A). Rv1438 may also influence host defense mechanisms through EGFR and UBC in both cell types.

Copper (Cu) is an essential element for the growth and development of most organisms, including bacteria. It has been shown that Cu in *Mtb* binds to various enzymes including cytochrome *c* oxidase and Cu/Zn superoxide dismutase. In addition, Cu helps *Mtb* resist oxidative stress (Piddington et al., 2001), suggesting that Cu is essential for *Mtb* survival. However, overload of Cu in most systems is toxic. A study reported that the concentration of Cu is dramatically increased in phagolysosomes of mouse Mϕs after infection with several *Mycobacterium* species (Wagner et al., 2005). Another study demonstrated that guinea pigs responded to *Mtb* infection by increasing Cu concentration in the lung lesions, and this coincides with a reduction in bacterial burden (Wolschendorf et al., 2011). These findings demonstrate that Cu is used by the host immune system to control *Mtb* infection, and *Mtb* evolves Cu homeostasis as a protective mechanism. The *rv0969* (*ctpV*) gene is part of a Cu-induced operon, and encodes a Cu-specific inner membrane efflux pump that transports excess Cu out of *Mtb* in order to prevent toxicity. In order to maintain Cu homeostasis in *Mtb*, it is possible that the bacteria has a repressor of Rv0969 in the absence of Cu. Rv0967 (CosR) represses the expression of *cso* (copper sensitive operon) and induces Cu in *Mtb* (Ward et al., 2008). As shown in Figure 6B, Rv0967 and Rv0969 are similarly expressed in Mϕs and DCs. This suggests that there may have been Cu homeostasis of *Mtb* during infection in both cell types. Rv0970 is an integral membrane protein, but its main function is currently unknown. The *rv0970* gene is regulated by several TFs in both cell types, specifically Rv0324 and Rv1423 in DCs and Rv0081 and Rv2324 in Mϕs, causing Rv0970 with no significant differential expression. This indicates that there are different regulatory functions of Rv0970 in Mϕs and DCs. In addition, Rv0970 interacts with Rv0967 and Rv1969 in both cell types, and may participate in Cu homeostasis. Thus, Rv0970 probably plays an important role in Cu homeostasis in both cell types, and controls different responses to different circumstances in Mϕs and DCs.

The interaction between bacteria–host occurs since their evolutionary origin and these mechanisms were implied in the evolution of the human (their homeostasis or their immune system). Most of bacteria that live with and within the human are part of the superorganism. There are different mechanisms employed by the host to limit the growth of *Mtb* such as nutrient and oxygen limitation, acidic pH, and formation of reactive oxygen/nitrogen intermediates, which forces *Mtb* to become dormant. Rv0081 is upregulated in multiple latency models, and is shown to be regulated by dormancy survival regulator (DosR) (Balázsi et al., 2008). DosR is essential for survival of *Mtb* during anaerobic dormancy, which mediates entrance into and throughout the dormant state (Leistikow et al., 2010). Another study demonstrated that the control of bacterial replication in latency in mice requires IFN-γ, TNF-α, and nitric oxide (NO) (Voskuil et al., 2003). Furthermore, the expression of Rv0081 could induce *Mtb* exposure to NO. These studies suggest that Rv0081 may play an important role in the dormancy of *Mtb*. However, the mechanism is not demonstrated in our HPCNs because at the 18 h time point, granulomas have not yet formed in Mϕs. Rv0081 has another role as a member of the ArsR/SmtB family of metal-dependent transcriptional regulators. As such, it functions as a regulator of metal homeostasis of *Mtb* for preventing metal stress within the host (Campbell et al., 2007). As shown in Figure 6B, Rv0081 has more specific PPI edges and regulatory genes in Mϕs, which could suggest that Rv0081 acts as a metal-dependent transcriptional regulator of *Mtb* specifically within Mϕs. Another TF, Rv0324, with an N-terminal HTH ArsR-type DNA-binding domain, may act as an ArsR metal-dependent transcriptional regulator (Li et al., 2015). Rv0324 has more specific PPI edges and regulatory genes in DCs, which could suggest that Rv0324 acts as a metal-dependent transcriptional regulator of *Mtb* specifically within DCs (Figure 6B). Thus, *Mtb* utilizes a different metal-dependent homeostasis pathway within the two cell types, which may result from the adaptive response that allows *Mtb* to survive in different circumstances.

Rv2234 (PtpA), a protein tyrosine phosphatase, has been shown to be an essential enzyme for the survival of *Mtb* in Mϕs (Koul et al., 2000). Furthermore, NO and ROS have been shown to reduce Rv2234 activity in Mϕs, and this disrupts the growth of *Mtb* in Mϕs (Ecco et al., 2010). In Figure 6B, the high expression of Rv2234 in Mϕs is not inhibited by NO or ROS. We also show that Rv2234 interacts with Rv0081 and Rv0324, which are both metal-dependent transcriptional regulators in Mϕs and DCs, respectively. This suggests that Rv2234 may be involved in the metal-dependent pathway in both cell types.

Rv1173 (FbiC) is essential for F_420_ production, and participates in the F_420_ biosynthetic pathway (Choi et al., 2002). F_420_ is catalyzed by F_420_-dependent glucose-6-phosphate dehydrogenase into H_2_F_420_ (Purwantini et al., 1997). One mechanism for host killing of *Mtb* is through phagosome acidification. The induction of NO in acidified phagosomes during activation of Mϕs, leads to its conversion to NO_2_. *Mtb* is more sensitive to NO_2_ than to NO under aerobic conditions, and has evolved a mechanism to decrease the antibacterial action of Mϕs by converting NO_2_ back to NO via H_2_F_420_ (Purwantini and Mukhopadhyay, 2009). Although NO is known to kill *Mtb* under both aerobic and hypoxic conditions, NO_2_ is more toxic than NO under aerobic conditions (Purwantini and Mukhopadhyay, 2009). The high expression of Rv1173 in *Mtb* in Mϕs compared to DCs, indicates greater nitrosative stress in Mϕs (Figure 4). The induction of Rv1173 increases the production of H_2_F_420_ to convert NO_2_ to NO for protecting *Mtb* from the nitrosative burst in Mϕs under aerobic conditions.

Rv1675c (Cmr) is a CRP/FNR family transcription factor that is expressed in response to cAMP levels (McCue et al., 2000). cAMP is a common second messenger molecule that plays an important role in catabolite repression, virulence, and signaling pathways in many bacterial pathogens including *Mtb* (McDonough and Rodriguez, 2012). During Mϕ infection, *Mtb* produces a cAMP burst within Mϕs to promote the survival of *Mtb* (Agarwal et al., 2009). In Figure 6B, high expression of Rv1675c in Mϕs reflects the high cAMP levels within Mϕs. Furthermore, Rv1675c interacts with many proteins, and has more specific edges in Mϕs than DCs. Rv1675c also interacts with three metal-dependent proteins Rv0081, Rv0324, and Rv0967 as previously mentioned. This indicates that Rv1675c may participate in the metal-dependent pathway that facilitates *Mtb* resistance to the induction of metal toxicity within Mϕs during early infection, although this has not yet been demonstrated. A recent study reported that Rv1675c regulates *rv1675c*, and is involved in Cmr-mediated gene regulation (Figure 6B; Ranganathan et al., 2015). In addition, the interaction of Rv1675c with Rv0667 may indirectly influence the host in HPCN (Figure 4). Altogether, Rv1675c may participate in *Mtb* host manipulation through Rv0667, and plays a role in protecting *Mtb* from metal toxicity by interaction with metal-dependent proteins in Mϕs. This suggests that Rv1675c is essential for *Mtb* survival during early infection in Mϕs, and could be a potential drug target.

Rv0353 is an *Mtb* antigen that can be processed and presented on MHC molecules, which promotes the production of antibodies against *Mtb*. It has been shown that Rv0353 favors the activation of DCs during early *Mtb* infection (Gupta et al., 2010). However, mir-636 inhibits *rv0353* in Mϕs (Figure 6B). In addition, the antigen processing capacity of Mϕs is reduced by *Mtb* proteins, causing a reduction in antibody production. In contrast, DCs can still process Rv0353 and present it on MHC molecules. Thus, repression of mir-636 and the *Mtb* membrane proteins, shown in Figure 6A, may recover the antigen processing capacity of Mϕs and increase the production of antibodies. In addition, Rv0353 also interacts with metal-dependent TFs including Rv0081 and Rv0324 (Figure 6B), indicating the participation of Rv0353 in metal-dependent homeostasis signaling pathways in both cell types.

#### Overview of the defensive mechanisms of the host and pathogen and the dysfunctions of the host in Mϕs and DCs

**Figure 7:**
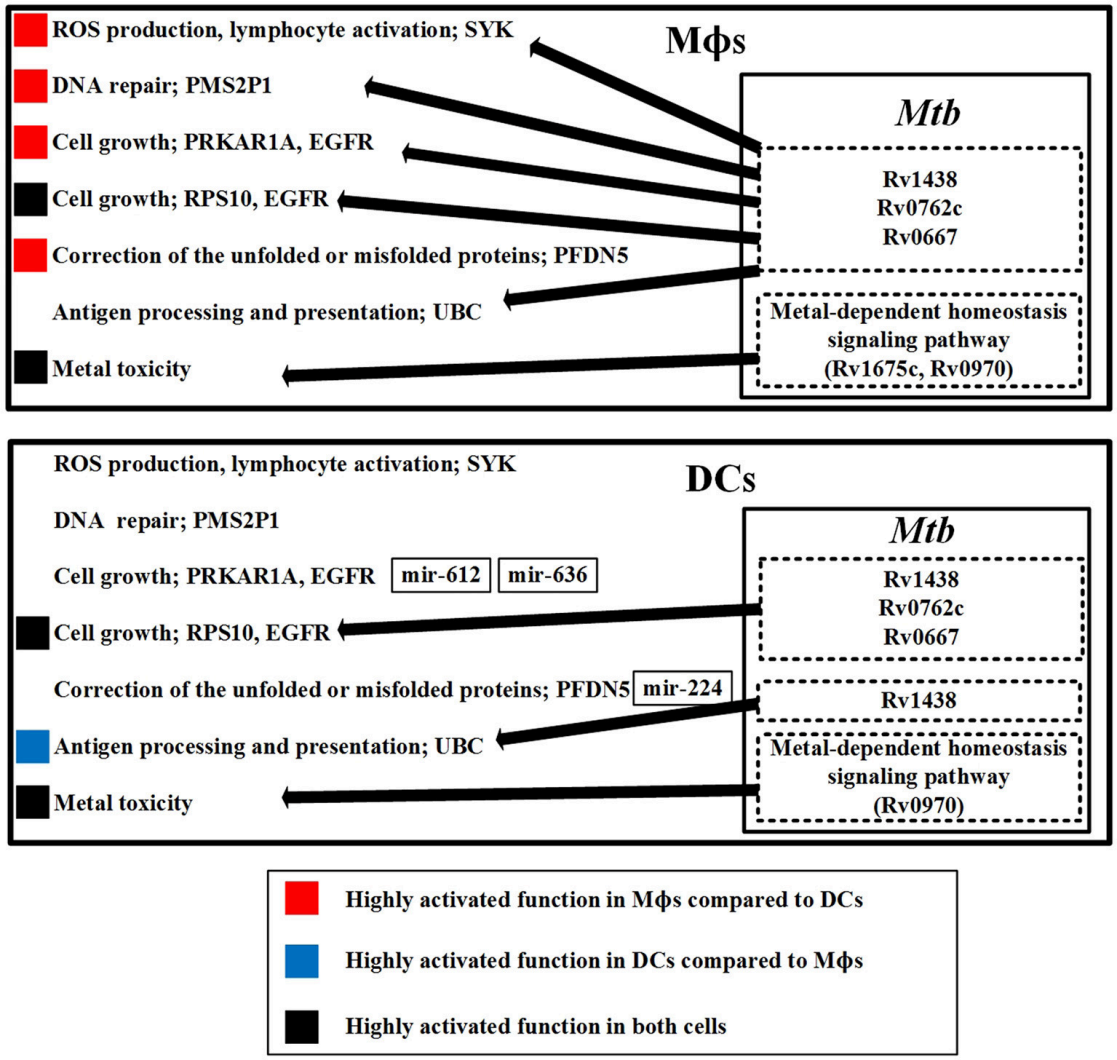
**The defensive mechanisms of the host and pathogen and the dysfunction of the host in Mϕs and DCs during early ***Mtb*** infection**. The upper figure summarizes the defensive mechanisms of the host in Mϕs during early *Mtb* infection. The defensive proteins (Rv1438, Rv0762c, and Rv0667) and metal-dependent homeostasis signaling pathway of *Mtb* within Mϕs counteract the host defense mechanisms. The lower figure summarizes the defensive mechanisms of the host in DCs during early *Mtb* infection. The defensive proteins (Rv1438, Rv0762c, and Rv0667) and metal-dependent homeostasis signaling pathway of *Mtb* within DCs influence host defense mechanisms. We observed that the defensive mechanisms of Mϕs are more easily influenced by *Mtb* than DCs, indicating that Mϕs are more susceptible to *Mtb* than DCs. In addition, the dysfunction in Mϕs such as DNA repair and cell growth caused by *Mtb* may easily cause the accumulation of mutations in Mϕs. Thus, *Mtb* infection of Mϕs may promote progression from TB to lung cancer.

Figure 7 provides an overview of the previously mentioned defense mechanisms in Mϕs and DCs. The diagram demonstrates that the same mechanisms employed by both cell types have different activity because of the influence from *Mtb* or as a result of miRNA inhibition.

First, we discuss the host mechanisms and the defensive *Mtb* proteins to counteract these host mechanisms. SYK is involved in ROS production, and is present only in Mϕs. It acts as an immune response in Mϕs to eliminate *Mtb*. However, SYK can be influenced by *Mtb* proteins, indicating that *Mtb* can interfere with ROS production in Mϕs. Another defense mechanism for host cells is metal toxicity, which is utilized by both cell types. However, *Mtb* evolves metal-dependent homeostasis signaling pathway to counteract the metal toxicity in both cell types.

The defensive mechanisms of the host can be counteracted by *Mtb*, causing dysfunction of host cells. PMS2P1 functions in DNA repair with its high expression in Mϕs, and can be influenced by *Mtb* proteins, causing DNA repair dysfunction in Mϕs. PRKAR1A is involved in cell growth and is highly expressed in Mϕs. Its expression is reduced in DCs because of mir-612 and mir-636 inhibition, which suggests that *Mtb* can easily dysregulate cell growth in Mϕs. RPS10 is also involved in cell growth, is present in both cell types, and can be influenced by *Mtb* proteins in both cell types. PFDN5 functions to correct unfolded or misfolded proteins. It is highly expressed in Mϕs and poorly expressed in DCs because of mir-224 inhibition. Without inhibition of PFDN5 by miRNA in Mϕs, the correction of unfolded or misfolded proteins would be constitutively active in Mϕs, which could contribute to progression from TB to cancer in Mϕs. UBC is involved in antigen processing and presentation, and can be influenced by *Mtb* proteins in both cell types. There are more *Mtb* proteins to interfere with UBC in Mϕs, which suggests that DCs are able to induce more antigen processing and presentation than Mϕs. *Mtb* induces cellular dysfunctions in DNA repair and cell growth in Mϕs. This suggests that *Mtb*-infected epithelial cells may have similar dysfunctions in DNA repair and cell growth, which may contribute to the progression of lung cancer.

### Drug targets, drug mining and multi-molecule drug design

A major preventative agent against TB is Bacille Calmette-Guérin (BCG), an attenuated vaccine strain extracted from ox infected with *M. bovis* by Calmette and Guérin. Bacille Calmette-Guérin vaccination is typically administered at birth, and is highly effective in preventing the development of TB. However, BCG efficacy decreases over time, and the protection in adults is not as effective as in children (Andersen and Doherty, 2005). Some studies have shown that BCG protects humans for only 10–20 years (Comstock et al., 1976; Hart and Sutherland, 1977; Sterne et al., 1998). Although several new drugs have been developed and are successful against tuberculosis, the development of multi-drug resistance tuberculosis (MDR-TB) and extensive drug resistance tuberculosis (XDR-TB) limit their efficacy (Johnson et al., 2006; Alexander and De, 2007). MDR-TB is resistant to isoniazid and rifampicin, which are the two most effective anti-TB drugs (Zignol et al., 2012). XDR-TB is also resistant to these as well as second-line drugs (Centers for Disease Control and Prevention, 2006).

Because of its important role in the defense against *Mtb* infection in Mϕs, we predicted Rv1675c as a potential drug target. Inhibition of Rv1675c may interfere with auto-regulation and the metal-dependent pathways of *Mtb* in Mϕs. Moreover, the cell wall or cell membrane proteins Rv1098c, Rv0967, Rv0667, Rv1696, and Rv2404c have been identified using automated two-dimensional, capillary high-performance liquid chromatography (LC) coupled with mass spectrometry (MS) (2DLC/MS) in TubercuList (Mawuenyega et al., 2005). In TubercuList, Rv1098c, and Rv0667 have been also identified as essential genes by Himar1-based transposon mutagenesis in H37Rv strain (Sassetti et al., 2003) while Rv1696 is required for growth in C57BL/6J mouse spleen, by transposon site hybridization (TraSH) in H37Rv (Sassetti and Rubin, 2003). However, in TubercuList, Rv0967 and Rv2404c have been identified as non-essential genes by Himar1-based transposon mutagenesis in H37Rv strain (Sassetti et al., 2003), and non-essential genes for *in vitro* growth of H37Rv, by sequencing of Himar1-based transposon mutagenesis (Griffin et al., 2011). Therefore, we suggested that Rv1098c, Rv0667, and Rv1696 are important for the survival of *Mtb* in both cell types and can be easily targeted by drugs. Because we have identified that the pathogen TF Rv0081 is positively affected by Rv0969 during the *Mtb* infection of Mϕs, the pathogen TFs Rv0081 and Rv0324 could be positively affected by Rv0969 during the *Mtb* infection of DCs. When Rv0969 is targeted by the proposed drugs, the expression levels of the pathogen proteins Rv1675c and Rv0970 could be attenuated during the *Mtb* infection of Mϕs and DCs (Figure 6). Therefore, the inhibition of drug targets Rv0967, Rv0969, and Rv0970 could lead to the inhibitive combination of Cu homeostasis during the *Mtb* infection of Mϕs and DCs. These proteins may also become potential drug targets for multi-molecule drug design for future therapy of *Mtb* infection. The repression of mir-636 and *Mtb* membrane proteins may help Mϕs increase antibody production. However, there is currently no drug to inhibit mir-636.

After predicting potential multiple drug targets, we then designed a potential multi-molecule drug for targeting these potential multiple drug targets through drug mining from literature review. To date, a drug database for drugs targeting *Mtb* proteins has not been constructed, so we explored studies predicting that some drugs may inhibit potential multiple drug targets. A study based on the comparison of binding sites of existing drugs for human use against the entire structural proteome of the pathogen predicted Lopinavir as a drug against the *Mtb* protein Rv1438 (TpiA) (Kinnings et al., 2010). Another study reported that TMC207 targets heavy metal P-type ATPases including Rv0969 (CtpV) (Andries et al., 2005; Novoa-Aponte and Soto Ospina, 2014), which may mediate *Mtb* P-type ATPase activation and enhance the metal toxicity to eliminate *Mtb* during metal burst in Mϕs and DCs. In addition, copper-boosting compounds ATSM and GTSM have been reported to increase the Cu level in *Mtb* rather than disrupting Cu homeostasis (Speer et al., 2013). Finally, we combine these drugs to generate potential multi-molecule drug as shown in Figure S5 for the potential multiple drug targets, which could be efficient for the treatment of *Mtb* infection in both cell types. The present drug molecule Lopinavir was predicted to target the *Mtb* protein Rv1438 (TpiA), which interrupts the interactions between Rv1438 and host proteins (Kinnings et al., 2010). Another study reported that the molecule TMC207 targets heavy metal P-type ATPase including Rv0969 (CtpV) (Andries et al., 2005; Novoa-Aponte and Soto Ospina, 2014), which may mediate *Mtb* P-type ATPase activation and enhance the metal toxicity to eliminate *Mtb* during metal burst in both cell types. In addition, copper-boosting compounds ATSM and GTSM have been reported to increase the Copper (Cu) level in *Mtb* instead of disrupting Cu homeostasis (Speer et al., 2013). Therefore, these three molecules could be combined as potential multi-molecule drug for the treatment in both Mϕs and DCs during early *Mtb* infection.

Furthermore, the results suggest that Rv1098c may help Rv0667, Rv0762c, and Rv1438 promote host-pathogen interactions and may help Rv0353 promote the production of antibodies against *Mtb*. When mir-636 has been suggested as a potential drug target to help Mϕs increase antibody production through the activation of Rv0353, Rv1098c plays an important role in increasing the antibody production in Mϕs. Although Rv1098c has been suggested as a potential drug target to help both cell types attenuate survival of *Mtb*, these two drugs that inhibit mir-636 and Rv1098c, respectively, may produce an inhibitory action to treat the *Mtb*-infected Mϕs.

## Conclusion

Tuberculosis is a global disease, accounting for almost 2 million deaths per year. Although, drugs such as isoniazid, rifampin, and pyrazinamide are used for curing tuberculosis, there is an urgent need to identify new drugs because of the presence of drug-resistant strains. Here, for the first time, we used a systems biology approach, big database mining, and microarray data to construct GWGEINs in both Mϕs and DCs during early *Mtb* infection. We identified differences in the defense mechanisms of the host and pathogen in Mϕs and DCs during early *Mtb* infection by analyzing HPCNs extracted from GWGEINs via the PNP method.

Except for the production of cytokines by the host, oxidative stress is another strategy used to kill pathogens. Oxidative stress was identified in Mϕs and DCs, but it was higher in Mϕs. However, the pathogen can influence the activity of SYK, which is involved in ROS production through interacting with UBC in Mϕs. Furthermore, ROS is also detrimental to the host since it could cause DNA damage (Tailleux et al., 2008). Therefore, *Mtb*-mediated dysfunction of DNA repair might contribute to the progression of lung cancer due to long-term accumulation of mutations in DNA and the interspecies interaction between Rv1438 and EGFR. In addition, *Mtb* can also influence the cell growth mediators RPS10 and PRKAR1A, through interacting with UBC in Mϕs. In contrast, DCs are less susceptible to *Mtb*. mir-612 inhibits the expression of PRKAR1A to reduce cell growth in DCs. DCs collect antigens as well as misfolded and unfolded proteins to form DALIS, which is then degraded at the late stage of DC maturation. Antigen peptides are then loaded onto MHC molecules and presented to B cells for antibody production. The difference in the defense mechanisms between *Mtb*-infected Mϕs and DCs can be observed in our HPCNs. Although, the role of antibody production is unclear as the control of *Mtb* requires cellular immunity, especially after antigen presentation by Mϕs, the genes involved in oxidative stress have been also reported to contribute to the difference in the defense mechanisms between *Mtb*-infected Mϕs and DCs (Tailleux et al., 2008).

Another strategy employed by the host to kill pathogens is the metal burst in *Mtb*. Although metals are needed for survival of the bacteria, the overload of metals becomes toxic. *Mtb* evolves adaptive mechanisms to overcome the excess or absence of Cu in *Mtb* in order to maintain Cu homeostasis. Rv0969 acts as an efflux pump to transport excess Cu out of *Mtb*, and Rv0967 represses the expression of *cso* in the absence of Cu. Furthermore, Rv0081 and Rv0324 are metal-dependent transcriptional factors that participate in the regulation of *rv0970*. Although, the function of Rv0970 is still unknown, we predict that it may play an important role in the metal-dependent homeostasis pathway. The difference between metal-dependent homeostasis pathways specific in Mϕs and DCs is also shown in HPCNs in Figure 6B. Although, the function of Rv1675c is still unknown, we found that it participates in the metal-dependent homeostasis pathway with higher expression and more specific interactions in Mϕs, indicating its important defensive role in Mϕs. Rv1675c could become a potential drug target. Another potential drug target is Rv1438, which interacts with the host proteins EGFR and UBC, and is essential for the survival of *Mtb*. The membrane proteins Rv1098c, Rv0967, Rv0969, Rv0970, Rv0667, Rv1696, and Rv2404c are important for the survival of *Mtb* in both cell types and can be easily targeted by drugs.

We observed that Mϕs are more susceptible to *Mtb* than DCs, and dysfunctions in Mϕs such as DNA repair, cell growth and the constitutive correction of unfolded or misfolded proteins may easily cause the accumulation of mutations in Mϕs. Thus, *Mtb*-induced dysfunctions in Mϕs suggest that the same dysfunctions may be present in *Mtb*-infected epithelial cells, and contribute to the progression of lung cancer.

Finally, we designed a potential multi-molecule drug to deal with the potential drug targets we proposed. However, because of the lack of a database that targets *Mtb* proteins, we explored several studies to deal with the essential protein Rv1438, the metal-dependent protein Rv0969, and the increase in Cu levels in *Mtb*, from which the multi-molecule drug was designed (Figure S5).

In the pathogen gene regulation model, we demonstrate that host-miRNA can inhibit pathogen-gene interactions. However, the influence of these pathogen-gene interactions on host-miRNA is still possible although it has not been yet been elucidated. Therefore, as novel cross-talk mechanisms between the host and pathogen are identified in the future, the models used in this study can be improved.

## Author contributions

CL, YL and BC: Data analysis and interpretation, manuscript writing, methodology development, conception and design, data analysis and interpretation, manuscript writing. All authors read and approved the final manuscript.

### Conflict of interest statement

The authors declare that the research was conducted in the absence of any commercial or financial relationships that could be construed as a potential conflict of interest.

## Abbreviations

AIC: Akaike information criterion
ANOVA: analysis of variance
APC: antigen presenting cell
APP: amyloid precursor protein
BCG: bacille Calmette-Guérin
CR: complement receptor
Cu: copper
DALIS: dendritic cell aggresome-like induced structures
DCs: dendritic cells
DC-SIGN: dendritic cell specific ICAM 3 grabbing non-integrin
DosR: dormancy survival regulator
GO: gene ontology
GRN: gene/miRNA regulation network
GWGEIN: cross-talk genome-wide genetic-and-epigenetic interspecies network
HPCN: host-pathogen core network
LAM: Lipoarabinomannan
MDR-TB: multi-drug resistance tuberculosis
MHC-II: major histocompatibility complex class II
miRNA: microRNA
Mtb: *mycobacterium tuberculosis*
Mϕ: macrophage
NCBI: National Center for Biotechnology Information
NO: nitric oxide
PKA: cAMP-dependent protein kinase
PNP: principal network projection
PPD: purified protein derivative
PPI: protein-protein interaction
PPINs: protein-protein interaction networks
RIα: type 1α regulatory subunit
RNIs: reactive nitrogen intermediates
ROS: reactive oxygen species
RPS10: Ribosomal protein S10
SLAM: signaling lymphocyte activation molecule
TF: transcription factor
Th1: T helper type 1
TLR: toll-like receptor
and XDR-TB: extensive drug resistance tuberculosis.
