# Supplementary material for: Genetic-and-Epigenetic Interspecies Networks for Cross-Talk Mechanisms in Human Macrophages and Dendritic Cells during MTB Infection
Source: Front Cell Infect Microbiol. 2016 Oct 18;6:124. doi: 10.3389/fcimb.2016.00124 (PMC5067469; doi:10.3389/fcimb.2016.00124)
Supplement: Supplementary file 1 [file Presentation1.PDF]

Supplementary Material

Investigating the Genome-wide Genetic-and-epigenetic Interspecies Networks for Cross-talk Mechanisms and Multi-molecule Drug Design in Human Macrophages and Dendritic Cells Both Infected with *Mycobacterium*

Cheng-Wei Li, Yun-Lin Lee, Bor-Sen Chen

*** Correspondence:** Bor-Sen Chen, PhD: bschen@ee.nthu.edu.tw

Supplementary Figures

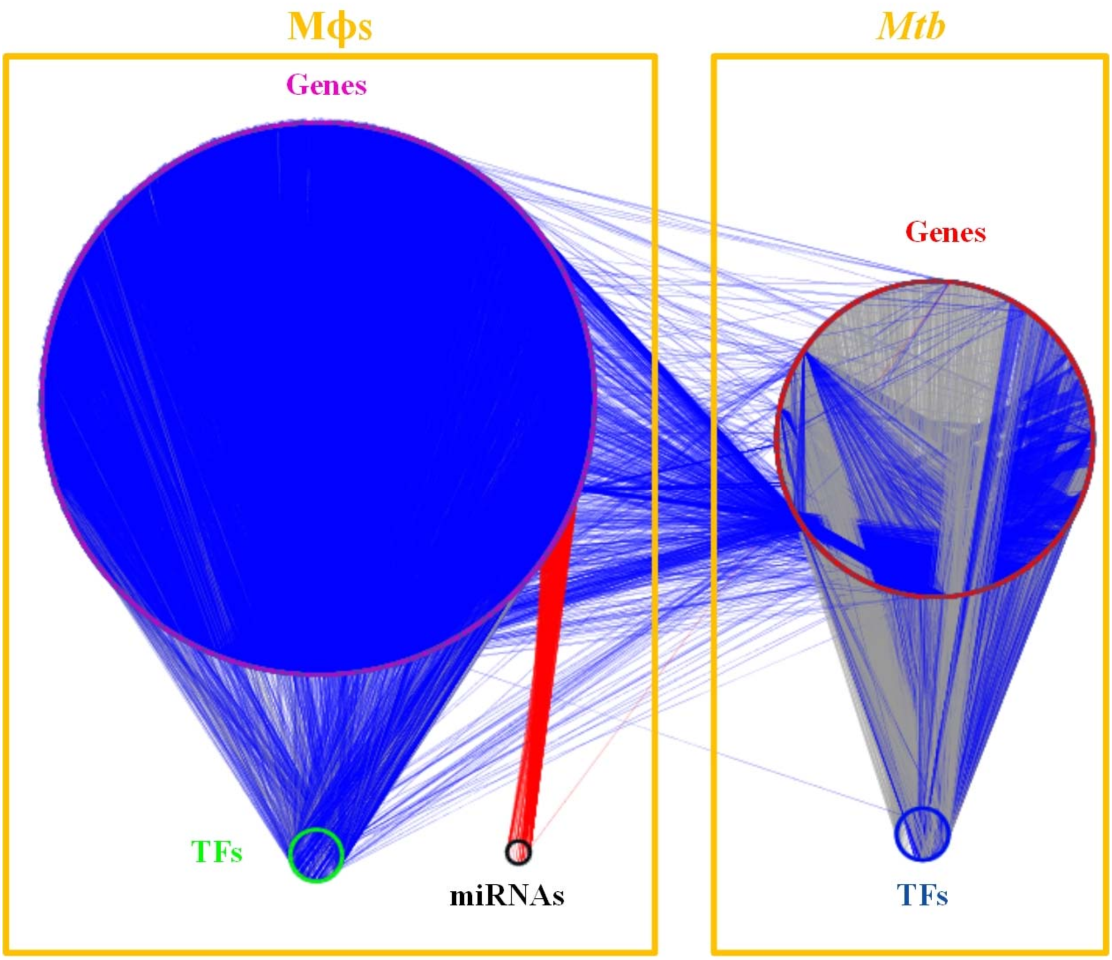

Figure S1. The real cross-talk GWGEIN in Mϕs. It represents the real genetic-and-epigenetic network connection of host and pathogen in Mϕs infected with *Mtb*. The edges in red represent the gene regulations of host-miRNA on host-genes or pathogen-gene. The edges in blue color represent the PPINs of host, pathogen and host-pathogen. The edges in grey represent the gene regulations of TFs on genes of host or pathogen.

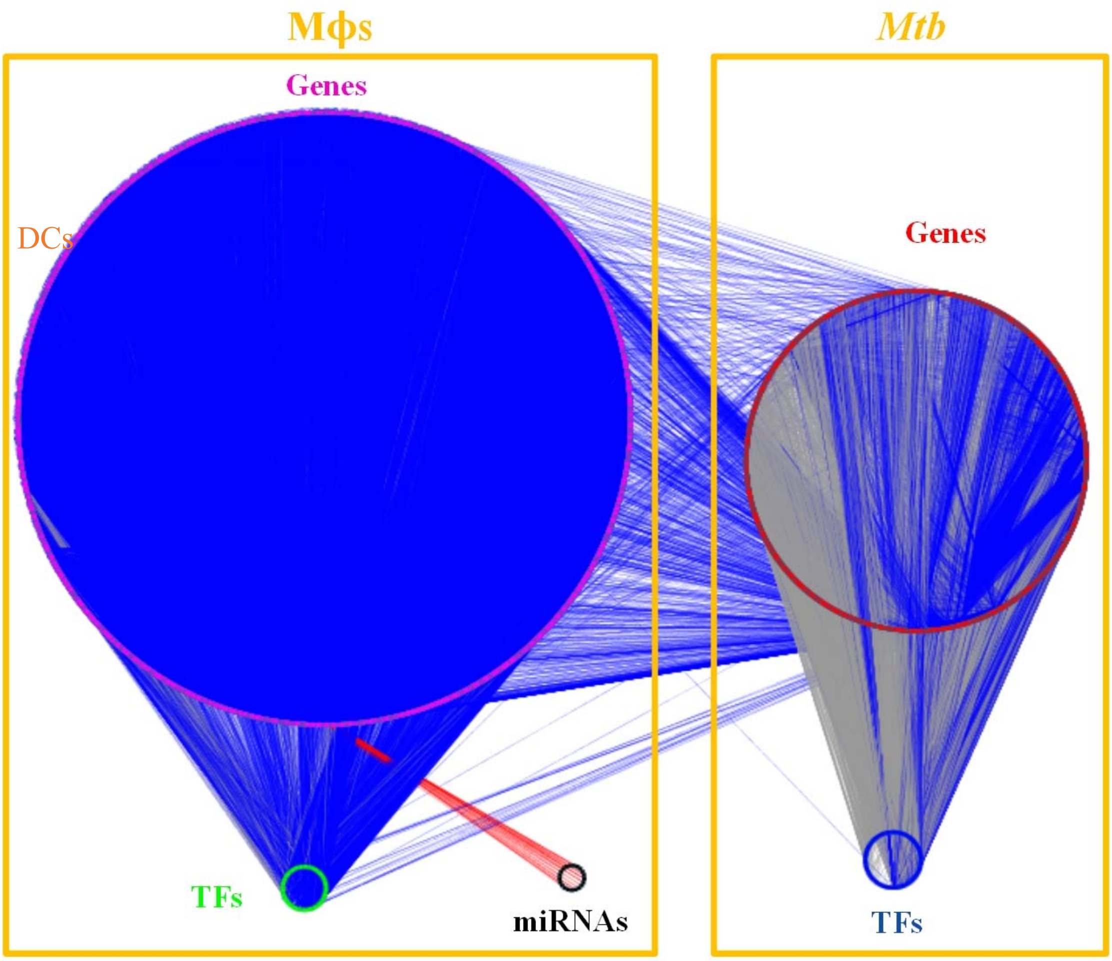

Figure S2. The real cross-talk GWGEIN in DCs. It represents the real genetic-and-epigenetic network connection of host and pathogen in DCs infected with *Mtb*. The edges in red represent the gene regulations of host-miRNA on host-genes or pathogen-gene. The edges in blue color represent the PPINs of host, pathogen and host-pathogen. The edges in grey represent the gene regulations of TFs on genes of host or pathogen.

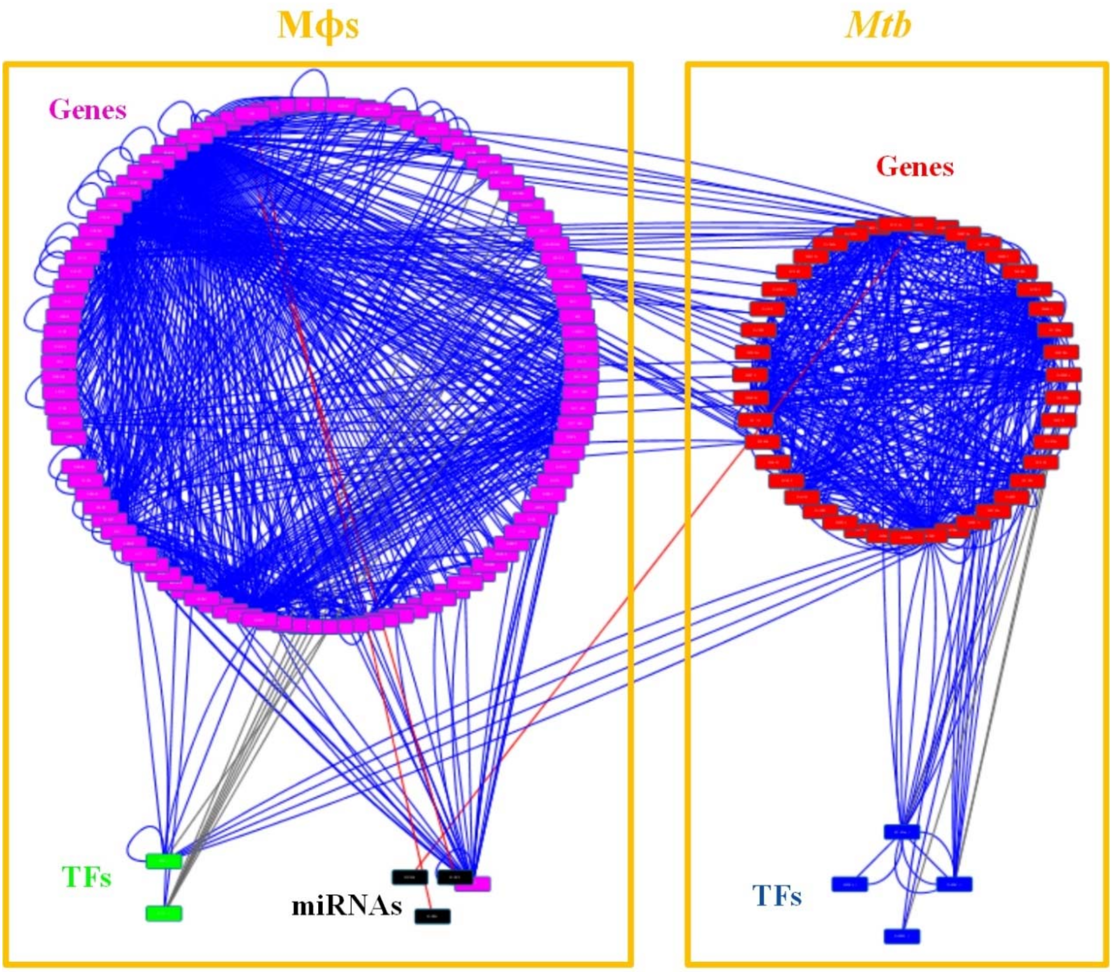

Figure S3. HPCN in Mϕs infected with *Mtb*. HPCN is extracted from GWGEIN in Mϕs during early *Mtb* infection by PNP. It represents the principal genetic-and-epigenetic network connection of host and pathogen in Mϕs infected with *Mtb*. The edges in red represent the gene regulations of host-miRNA on host-genes or pathogen-gene. The edges in blue represent the PPINs of host, pathogen and host-pathogen. The edges in grey represent the gene regulations of TFs on genes of host or pathogen.

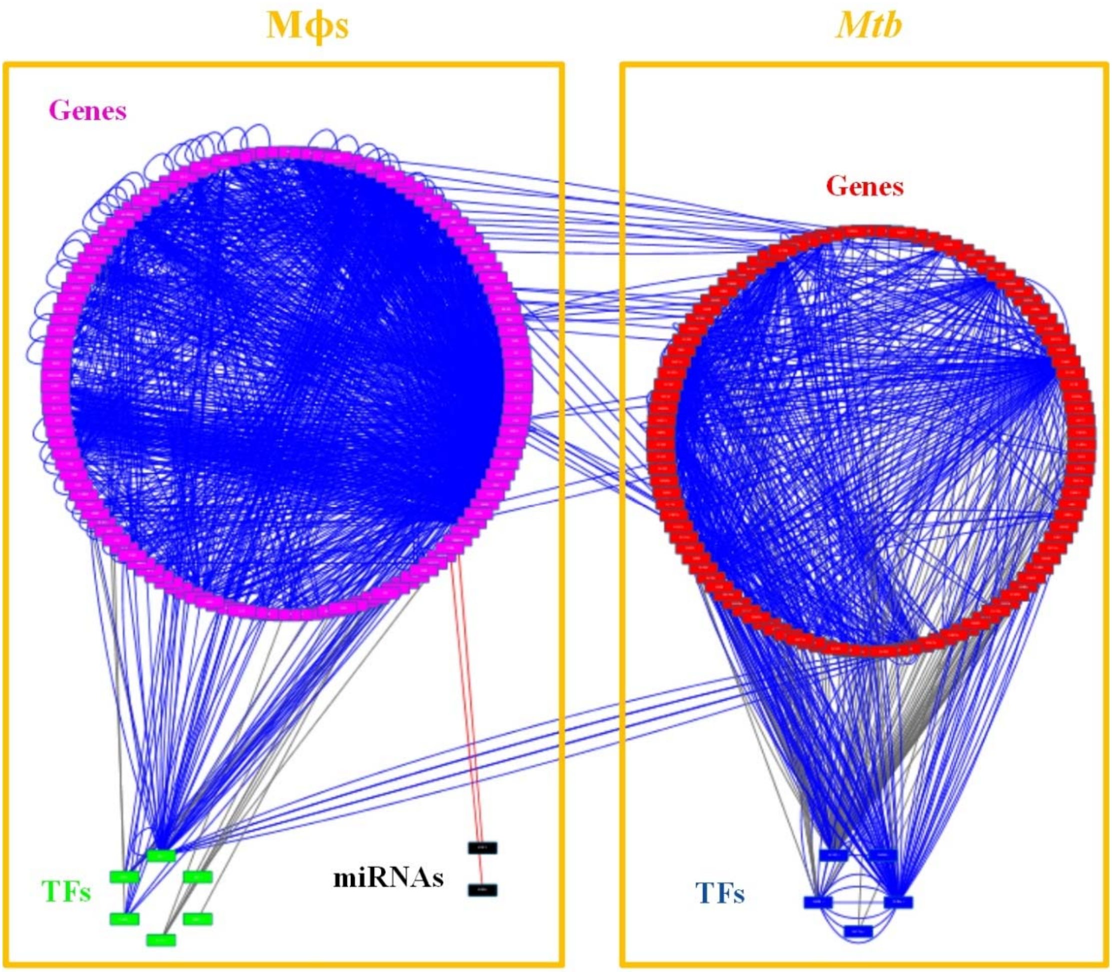

Figure S4. HPCN in DCs infected with *Mtb*. HPCN is extracted from GWGEIN in DCs during early *Mtb* infection by PNP. It represents the principal genetic-and-epigenetic network connection of host and pathogen in DCs infected with *Mtb*. The edges in red represent the gene regulations of host-miRNA on host-genes or pathogen-gene. The edges in blue represent the PPINs of host, pathogen and host-pathogen. The edges in grey represent the gene regulations of TFs on genes of host or pathogen.

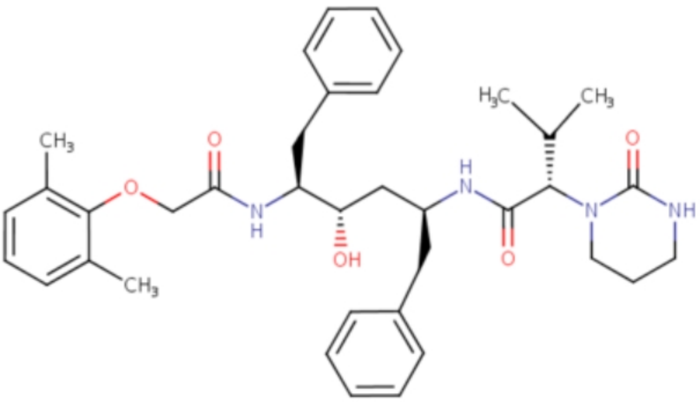

Lopinavir

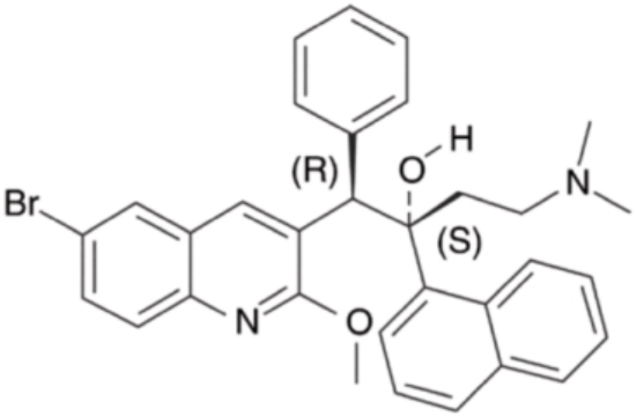

TMC207

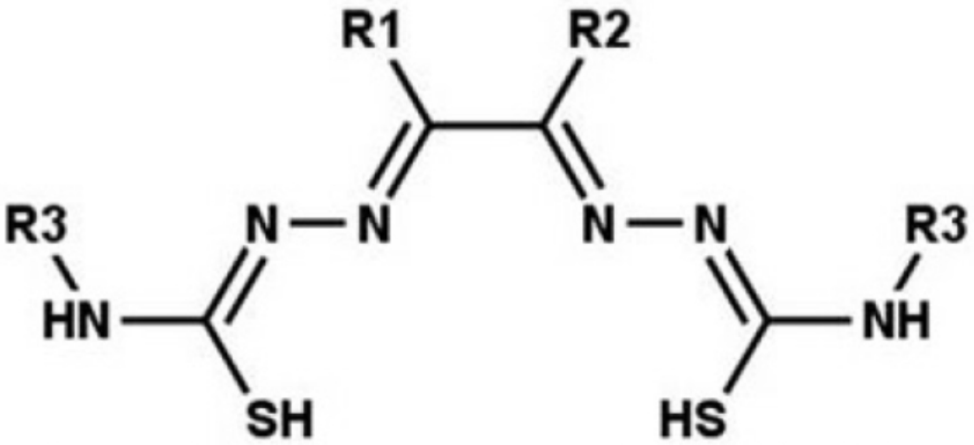

Copper-boosting compounds ATSM and GTSM

Figure S5. The multi-molecule drug for the potential multiple drug targets
